# A Native Bioactive Interface Functionalized with Osteoprogenitor Stem Cell-Derived Migrasomes for Enhanced Bone Regeneration

**DOI:** 10.34133/research.1220

**Published:** 2026-03-30

**Authors:** Hongming Zhang, Jiajia Wang, Rong Yang, Xinyu Song, Rongpu Liu, Junyi Wang, Lingxi Meng, Zhuoran Xu, Ilya A. Vinnikov, Guangzheng Yang, Wenjie Zhang

**Affiliations:** Department of Prosthodontics, Shanghai Ninth People’s Hospital, Shanghai Jiao Tong University School of Medicine; College of Stomatology, Shanghai Jiao Tong University; National Center for Stomatology; National Clinical Research Center for Oral Diseases; Shanghai Key Laboratory of Stomatology; Shanghai Research Institute of Stomatology, Shanghai, China.

## Abstract

The regeneration of large bone defects remains a major clinical challenge due to the lack of stable and effective osteoinductive signals. Although extracellular vesicles have shown promising potential for cell-free bone regeneration, their application is largely constrained by complex purification and embedding into scaffolds. Migrasomes, newly identified organelles with the extracellular matrix affinity, represent a promising yet underexplored avenue for cell-free tissue engineering. Here, we report a migrasome-enriched bioactive layer as a functional osteoinductive interface for cell-free bone regeneration. We demonstrated that osteoprogenitor stem cells (OPSCs) derived from human cortical bone exhibit robust osteogenic capacity and, upon osteogenic induction, promote the release and deposition of migrasomes together with calcium on culture surfaces. Utilizing these characteristics, we developed an in situ deposition strategy where OPSCs are preseeded on biphasic calcium phosphate (BCP) scaffolds, induced to mineralize, followed by decellularization. This process robustly preserves a native, osteogenic migrasome layer on the scaffold without the need for vesicle isolation or chemical conjugation. The resulting migrasome-functionalized scaffolds markedly up-regulated osteogenic gene expression and promoted bone regeneration in a murine calvarial defect model. Altogether, these findings validate migrasomes as a potent, endogenous signaling platform for bone tissue engineering. Moreover, our new paradigm for cell-free biomaterials employing cellular secretomes opens a new frontier in regenerative medicine, where the transient activity of cells is permanently captured to direct tissue repair.

## Introduction

The repair of critical-size bone defects remains a major clinical challenge due to the limited self-healing capacity of bone tissue [1]. This challenge is particularly pronounced in dental implantology [2], where insufficient bone volume often compromises implant stability and long-term success [3]. It is estimated that over 2 million bone augmentation surgeries are performed annually worldwide, driving the global bone graft substitutes market to exceed 2.5 billion dollars per year [4]. While autologous bone grafting remains the clinical gold standard, its application is constrained by donor site morbidity and limited availability. Consequently, biomaterial-based bone graft substitutes have been widely adopted. However, they are impeded by a fundamental bottleneck: most clinically used biomaterials lack endogenous osteoinductive cues capable of orchestrating host cells toward organized bone formation [5].

Recent advances in regenerative medicine have highlighted the potential of cell-free therapies as a promising solution [6]. Cell-free therapies leverage cell-derived instructive cues such as extracellular vesicles to activate endogenous regeneration without transplanting living cells. Compared with direct cell transplantation, these acellular approaches effectively circumvent issues such as poor cell survival, limited engraftment and immunogenicity [7–9].

Migrasomes, a recently discovered class of extracellular vesicle-like organelles [10], represent a new frontier in cell-derived bioactive components [11]. They form at the termini of retraction fibers during cell migration, adhere to the extracellular matrix (ECM) via integrins, and can carry diverse biological cargo, including RNAs [12], proteins [13], lipids [14] and mitochondria [15]. A key finding is that migrasome contents and signaling activity are dictated by the type and physiological state of their parental cells [16]. For example, macrophage-derived migrasomes promote fracture healing by inducing osteogenic differentiation of bone marrow stem cells (BMSCs) [17,18]. Neutrophil-derived migrasomes adsorb and enrich coagulation factors to participate in hemostasis [19], whereas monocyte-derived migrasomes accumulate vascular endothelial growth factor and chemokines during embryonic development to guide angiogenesis [20]. Moreover, even migrasomes from the same cell type (e.g., monocytes) can exert divergent biological functions; in cerebral amyloid angiopathy, they activate complement-dependent pathways that disrupt the blood–brain barrier [21]. These characteristics of migrasomes delineate their capacity to closely recapitulate the functional state of their parental cells.

Despite growing interest in the biological functions and biomedical applications of migrasomes, their practical utilization remains limited by the reliance on detachment and density-gradient centrifugation [22]. Although surface engineering approaches, such as titanium dioxide nanotube substrates, can enhance migrasome production [23], the fundamental constraint persists: isolation and reapplication disrupt the native spatial organization and compositional integrity of migrasomes, thereby compromising their translational utility. Notably, migrasomes remain highly stable and immobile after breaking away from the retraction fibers [24]. This property inspired a paradigm shift: rather than treating migrasomes as entities requiring their isolation and purification, we conceptualized them as a bioactive “imprint” of a desired cellular state that could be directly enriched onto a scaffold surface.

**Fig. 1.**
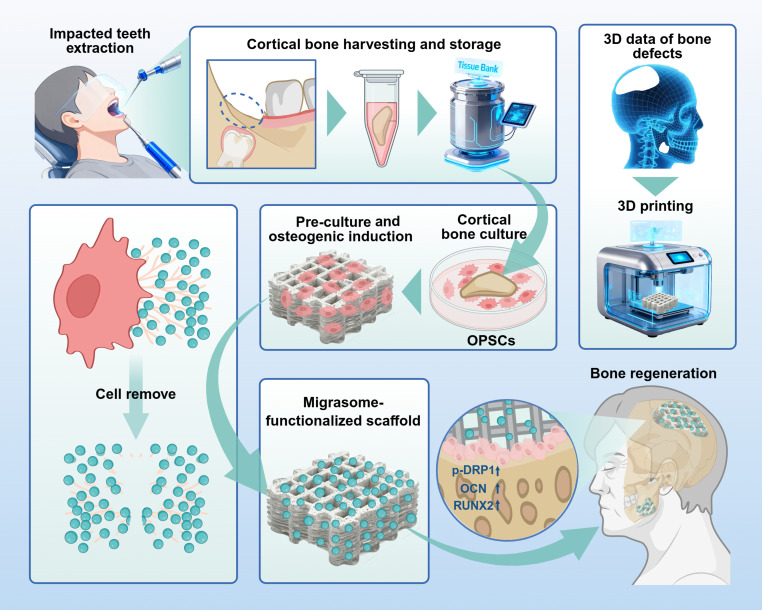
Bioactive biphasic calcium phosphate scaffolds functionalized with osteoprogenitor stem cell-derived migrasomes for enhanced bone regeneration. Cortical bone tissue obtained from impacted tooth extraction is preserved in a tissue bank. When needed, osteoprogenitor stem cells (OPSCs) are acquired through in vitro culture and preseeded on biphasic calcium phosphate (BCP) scaffolds customized to match the shape and size of the bone defect. Following cell removal, native migrasomes remain on the surface, resulting in a migrasome-functionalized scaffold ready for immediate in vivo bone defect repair and regeneration. p-DRP, phosphorylat4ed dynamin-related protein 1 (dynamin 1 like); OCN, osteocalcin (bone γ-carboxyglutamate protein); RUNX2, RUNX Family Transcription Factor 2.

In this study, we cultured human cortical bone-derived osteoprogenitor stem cells (OPSCs) and applied osteogenic induction (OI) to promote the in situ deposition of migrasomes onto biphasic calcium phosphate (BCP) scaffolds. Following optimized decellularization, this strategy yielded scaffolds coated with a native bioactive layer, bypassing the need for vesicle purification or chemical conjugation. Using this strategy, we demonstrated that migrasome-functionalized scaffolds enhance osteogenic differentiation and bone regeneration. Conceptually, this work reframes extracellular vesicles from drugs to interfaces: rather than delivering vesicles to tissue, we enable tissue-specific vesicles to construct the interface that instructs the tissue. Thus, we introduce a biomimetic platform that redefines the application paradigm for extracellular vesicles in tissue engineering, establishing establish migrasomes as a new class of bioactive interfaces for cell-free bone repair (Fig. 1).

## Results

### Cortical bone-derived cells migrate through canaliculi in vitro

**Fig. 2.**
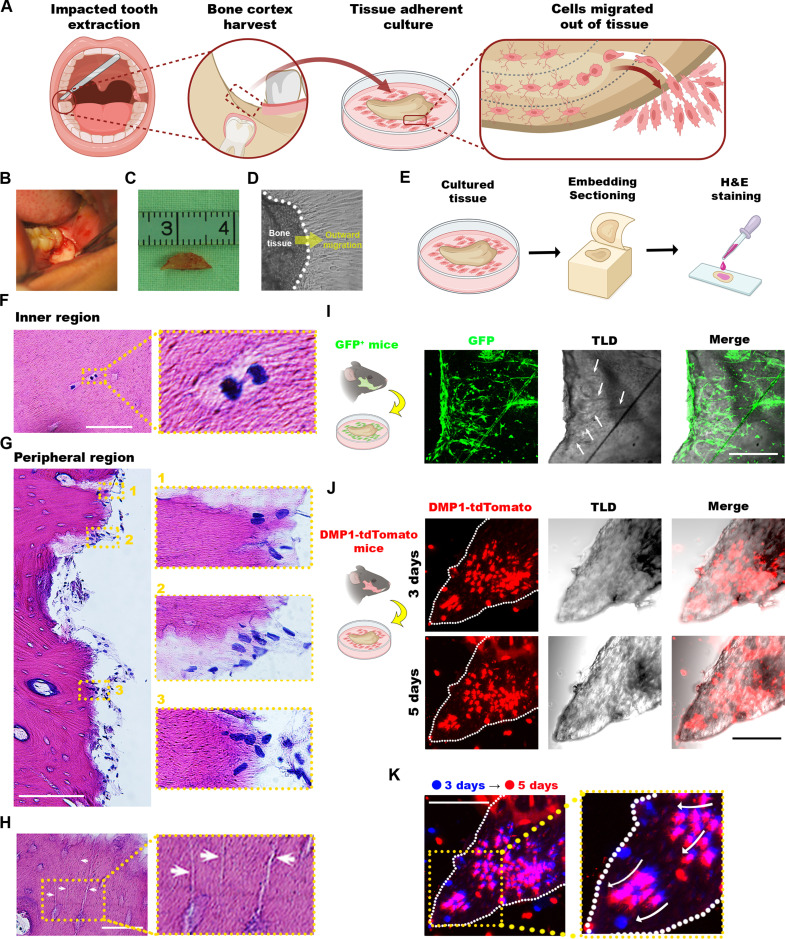
Active outward migration of cortical bone-derived cells. (A to D) Schematic illustration of cell migration in the mandibular cortical bone harvest from the buccal or occlusal region of the third molar (A), representative macrophotographs of the harvest sites (B), dimensions of harvested cortical specimens (C), and microphotographs of outward migration of spindle-shaped cells from the cortical bone after 7-d in vitro culture (D). (E) A scheme illustrating the preparation of the adherent-cultured cortical bone for histological examination. (F to H) Representative microphotographs of an inner region after 3-d culture revealing actively dividing cells (F), a peripheral region featuring outward migration (1, cells within inner edge; 2, cells spanning cortical margin; 3, cells beyond cortical edge) after 7-d culture (G), and canaliculi (arrows) spanning lacunae to cortical periphery (H) within the hematoxylin and eosin (H&E)-stained adherent-cultured bone. (I to K) Representative fluorescent microphotographs of green fluorescent protein-positive (GFP^+^) cells derived from mice arranged within and along the bone edges, featuring an outward extension tendency aligned (arrows) with the canaliculi (I), the distribution of dentin matrix acidic phosphoprotein-positive (DMP1^+^) cells after 3 and 5 days of in vitro culture (J) and outward migration of cells 3 (blue) to 5 (red) d after culturing (K). Scale bars in μm: 50 (H) and 100 (all other panels). TLD, Through-the-Lens Detector imaging.

Clinically, combining cortical bone fragments with bone graft materials in guided bone regeneration yields superior outcomes compared to bone grafts alone, a phenomenon that inspired our exploration of cells emigrating from cortical bone (Fig. 2A). Following isolation and removal of the gingiva and periosteum, cortical bone fragments obtained from the occlusal or buccal cortical plate removed during extraction of impacted mandibular third molar were cultured in vitro (Fig. 2B and C). After 7 days, the fragments adhered to the culture dish, with a large number of spindle-shaped cells migrated out of the tissue (Fig. 2D). Consistent with our previous studies demonstrating that even after cryopreservation, cortical bone yields a substantial population of osteogenic cells capable of promoting long-term repair of segmental defects of alveolar and jawbone [25,26], we sought to delineate the characteristics and migration pathways of these emigrated cells from the osteocyte-rich dense cortical bone matrix. Histological analysis of cultured cortical bone revealed active cell division within the inner tissue regions by day 3, followed by a progressive outward migration from the cortical margins by day 7 (Fig. 2E to G). Interestingly, the bone cortex featured numerous canaliculi (Fig. 2H), suggesting a potential migratory channel. To directly investigate this relationship, we evaluated mandibular cortical bone from transgenic mice and demonstrated that spindle-shaped GFP^+^ cells strongly colocalized with abundant canaliculi traversing from the cortical interior to the periphery (Fig. 2I). This result further suggests that these canaliculi might serve as migratory channels. Next, given the important role of dentin matrix protein 1 (DMP1) in osteoblast-driven mineralization [27], we cultured cortical bone from DMP1-cre; tdTomato mice to track osteogenic cell dynamics. Longitudinal immunofluorescence imaging revealed that DMP1^+^ cells were initially distributed both inside and outside the tissue (Fig. 2J) but progressively migrated toward the tissue margin over time (Fig. 2K). Altogether, these findings demonstrate that cortical bone-derived cells actively proliferate and induce directed outward migration via the lacunocanalicular network.

### Osteoprogenitor stem cells generate osteogenic organoids

**Fig. 3.**
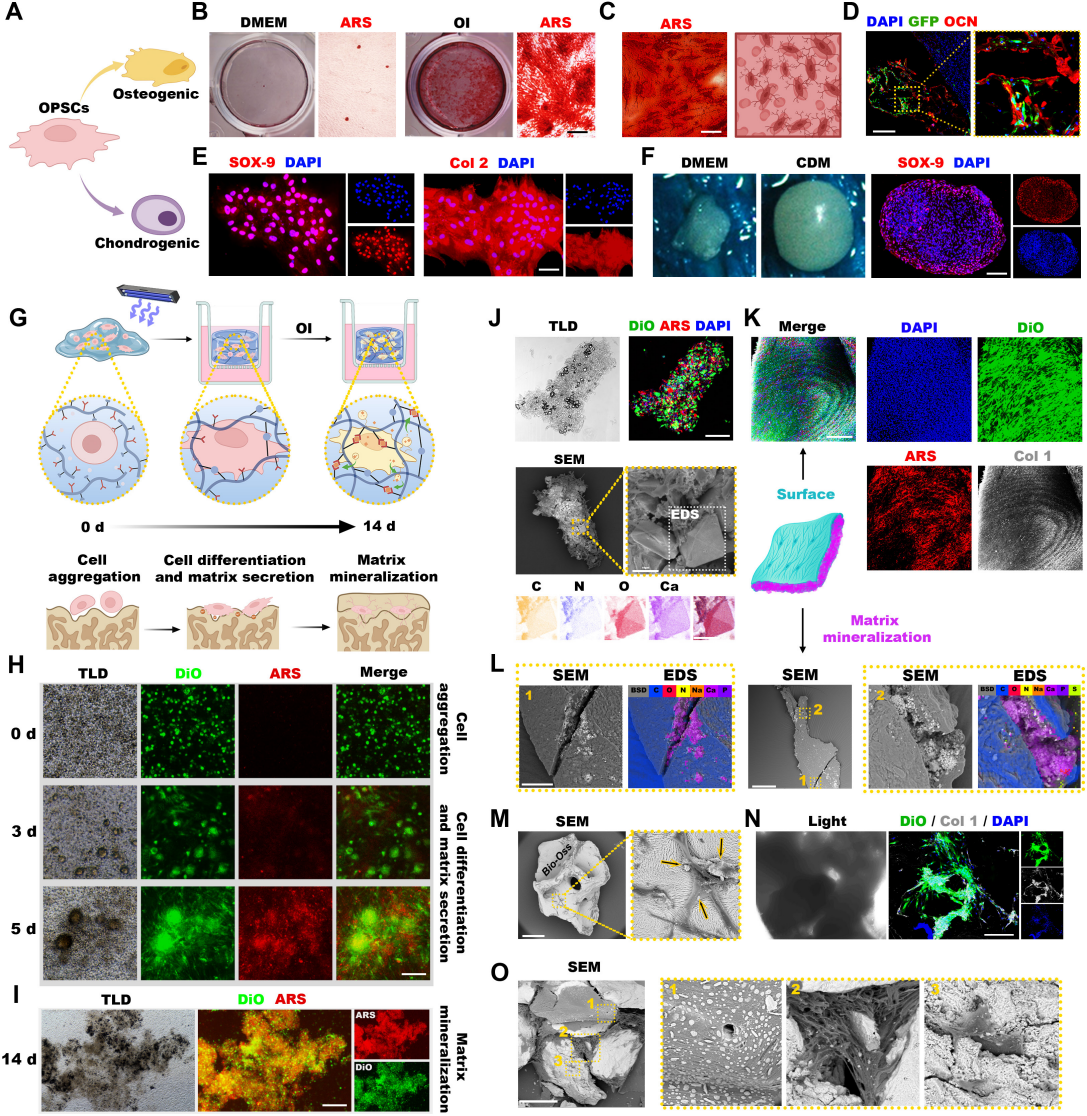
Osteogenic organoid engineering via intrinsic osteogenesis and mineralization capacities of OPSCs. (A) Schematic illustration of OPSC differentiation paths. (B and C) Representative photographs of alizarin red S (ARS)-stained OPSCs on day 14 of osteogenic induction (OI) featuring extensive mineralization compared to controls depicted in the left images (B) and on day 28 of OI, when the cell membranes exhibited extensive elongation with antenna-like projections and secreted abundant mineralized deposits around the cells, resulting in the formation of bone-like tissue structures, similar to that shown in the right illustration (C). (D) The tissue immunofluorescence staining showed that OPSCs-GFP exhibited high expression of OCN. (E) OPSCs were cultured in a flat dish for 14 days under chondrogenic induction conditions. Immunofluorescence staining showed high expression of SOX-9 and Col 2. (F) After 21 days of chondrogenic induction in a 3D culture, the cells formed cartilage nodules, and immunofluorescence staining revealed high expression of SOX-9. (G) Schematic of hydrogel crosslinking encapsulating OPSCs and subsequent osteogenic organoid formation. (H) DiO and ARS staining of OPSCs in hydrogel during OI (days 0, 3, and 5). (I) After 14 days of induction, the cells continued to aggregate, and the hydrogel began to curl up, forming a clumpy and wispy structure. (J) Immunofluorescence showed that calcium salt-like deposits were surrounding the cells. Scanning electron microscopy (SEM) and energy-dispersive x-ray spectroscopy (EDS) mapping showed the structure of organoid and the deposition of O and Ca elements. (K) Following 4 weeks of culture, the hydrogel coalesced into an integrated osteogenic organoid with a dense structure. Immunofluorescence images showed cells, mineralized nodules, and Col 1 co-constituting the structure of the surface. (L) SEM revealed that the inner part contained a large amount of mineralized matrix. (M) SEM images of OPSCs cultured on Bio-Oss bone grafts at day 7. (N) Immunofluorescence images showed the cells fully extend and produce Col 1. (O) Day 28 SEM showed cellular bridging of Bio-Oss bone grafts with extensive mineralization (1, small calcium deposits; 2, large calcium deposition; 3, cell-mediated particle integration). Scale bars in µm: 500 (I), 300 (M and O), 200 (B, D, F, H, and K), 100 (N), 50 (C, E, J, top and middle, and L), and 10 (J, bottom).

Upon migration and subsequent passaging, the OPSCs exhibited a typical fibroblast-like, spindle-shaped morphology and a parallel migratory aligned pattern, characteristic for mesenchymal stem cells (Fig. S1). Accordingly, immunofluorescence staining images confirmed the robust expression of canonical stem cell surface markers, such as STRO-1, CD29, CD90, CD73, and CD105, but not hematopoietic markers CD34 or CD45 (Fig. S2). We next evaluated multipotency of the OPSCs (Fig. 3A). Following osteogenic induction (OI), the cells generated abundant calcium deposition by day 14 (Fig. 3B), which progressively evolved into extensive bone-like mineralized structures by day 28 (Fig. 3C). To validate their in vivo osteogenic capacity, GFP-labeled, preinduced cells were seeded onto gelatin sponges and implanted subcutaneously into nude mice. By week 2, fluorescence analysis confirmed robust cell survival and expression of the late osteogenic marker osteocalcin (OCN, Fig. 3D). Furthermore, chondrogenic induction evoked a time-dependent up-regulation of SOX-9 and type II collagen (Col 2, Fig. S3). Indeed, in both planar (Fig. 3E and Fig. S4) and 3-dimensional (3D) cultures, induced OPSCs, but not uninduced controls, formed tightly connected cell aggregates and distinctive cartilage nodules characterized by widespread SOX-9 and Col 2 expression (Fig. 3F). Consistently, subcutaneous implantation of OPSCs combined with TGF-β3 generated dense, cartilage-like tissue featuring reduced vascularization and enriched Col 2 deposition (Fig. S5). Interestingly, while these cells exhibited relatively weak adipogenic potential (Fig. S6), they demonstrated stronger osteogenic mineralization (Fig. S7), and enhanced proliferative capacity (Fig. S8) compared to conventional bone marrow mesenchymal stem cells (BMSCs). Based on this pronounced osteogenic bias and robust proliferative profile, we defined this population as osteoprogenitor stem cells (OPSCs).

To validate the capacity of these cells to generate structurally competent mineralized tissues, we cultured them within a biomimetic 3D dual-crosslinked sodium alginate hydrogel. OPSCs were placed in the upper chamber of a transwell filled with the culture medium in both chambers. The initial lithium phenyl-2,4,6-trimethylbenzoylphosphinate (LAP)- and ultraviolet (UV) light-mediated crosslinking was followed by dynamic release of calcium ions (Ca^2+^) by differentiating OPSCs to promote secondary ionic crosslinking with glucuronic acid residues of the sodium alginate hydrogel, effectively integrating cellular biomineralization with structural maturation (Fig. 3G). Longitudinal imaging of alizarin red S (ARS)- and 3-octadecyl-2-[3-(3-octadecyl-2(3H)-benzoxazol-2-ylidene)-1-propen-1-yl] benzoxazole perchlorate (DiO)-labeled osteogenically induced OPSCs revealed dynamic mineralization. Indeed, while uniformly dispersed initially, by day 3 of OI, the cells rapidly extended and aggregated into discrete red fluorescence-positive clusters. These grew larger by day 5 driven by cellular proliferation, with enhanced crosslinking at the mineralization sites and abundant red nodular fluorescence both inside and around the clusters (Fig. 3H). Strikingly, after weeks of continuous OI, the constructs matured into highly polymerized layered organoids. By week 2, gradual aggregation of cells into small clusters and mineralization of the matrix led to the formation of a dense outer cellular layer enveloping a highly mineralized calcium matrix core (Fig. 3I). The hydrogel surface revealed the cells infiltrating and embedded within a porous, regular crystalline-like matrix predominantly composed of carbon, oxygen, and calcium (Fig. 3J). With calcium deposits revealing further mineralization by week 4 of continuous OI, the matrix core was surrounded by type I collagen (Col 1)-rich dense layer (Fig. 3K). The intricate architecture of this lamellar structure featured the outer layer predominantly composed of carbon, and the inner calcium-rich core (Fig. 3L).

Given the profound bone-forming capacity of OPSCs within these organoids, we next evaluated their ability to mineralize clinically relevant bone substitute materials such as Bio-Oss. By day 7, OPSCs robustly adhered to and spread on the graft surfaces promoting early mineralization and extensive Col 1 expression around the calcium deposits (Fig. 3M and N). Following 28 days of culture, the cells evoked profound mineralization, generating both small and large calcium deposits while concurrently migrating to bridge adjacent bone grafts (Fig. 3O). Altogether, these results demonstrate that OPSCs possess robust and versatile osteogenic differentiation capacity across 2D, 3D, and clinically applied biomaterial microenvironments, efficiently directing the formation of structurally organized, mineralized tissue constructs.

### OPSCs release pro-osteogenic migrasomes after OI

**Fig. 4.**
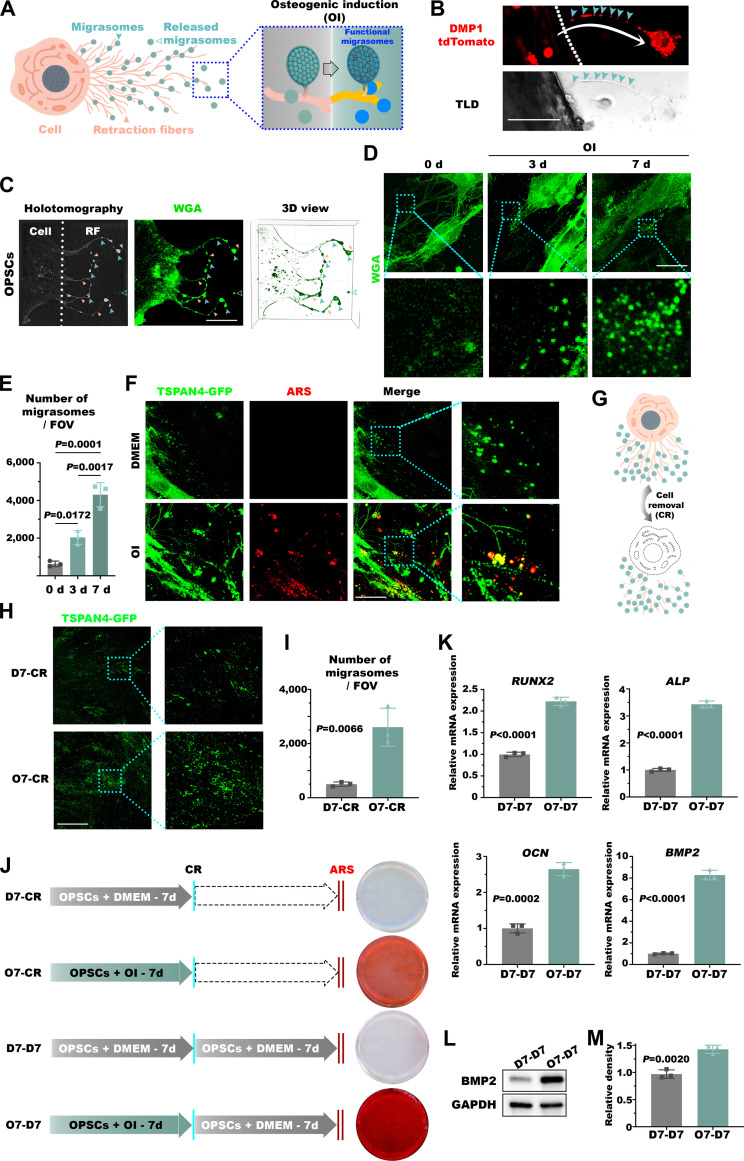
OPSCs release pro-osteogenic migrasomes during osteogenic differentiation. (A to C) Schematic illustration (A) and representative fluorescent (B) and holotomographic (C) microphotographs of migrasomes released by OPSCs, featuring retraction fibers (RF) of migrasomes at the trailing edges of cells during in vitro culture of cortical bone. Wheat germ agglutinin (WGA)-stained migrasomes (cyan arrows) are connected to OPSCs via retraction fibers (pink arrows). (D and E) Representative fluorescent microphotographs of WGA-stained migrasomes produced by OPSCs at varying OI time points (D) and quantification of migrasomes per each field of view (FOV) (E). (F) Representative fluorescent microphotographs demonstrating co-localization of the calcium salt-like deposits and the migrasomes upon OI. TSPAN4, tetraspanin 4. (G to I) Schematic illustration (G), representative fluorescent microphotographs (H), and quantification (I) indicating featuring increased migrasome retention after cell removal (CR) following 7 days of OI (O7-CR) compared to their uninduced counterparts cultured in DMEM for 7 days followed by decellularization (D7-CR). (J) Representative microphotographs of ARS-stained samples with the following treatments: decellularization after 7 days of DMEM culture (D7-CR), OI for 7 days prior to decellularization (O7-CR), decellularization after 7 days of DMEM culture followed by re-seeding naive OPSCs in DMEM for 7 days (D7-D7), and decellularization after 7 days of OI followed by re-seeding naive cells in DMEM for 7 days (O7-D7) with the latter sample demonstrating the most abundant calcium deposition, some deposition revealed in the O7-CR, but not D7-CR or D7-D7 samples. (K) Quantitative real-time polymerase chain reaction (qRT-PCR) demonstrating upregulated expression of osteogenic genes in O7-D7 samples compared to uninduced D7-D7 controls. ALP, alkaline phosphatase; BMP2, bone morphogenic protein 2. (L and M) Representative Western blot (L) and quantification (M) indicating elevated BMP2 protein in O7-D7 samples compared to D7-D7 controls. Scale bars in µm: 50 (B, D, F, and H) and 20 (C).

During the culture and characterization of OPSCs, we observed the presence of migrasomes in their vicinity. Given the robust osteogenic capacity of OPSCs, we further investigated the characteristics and potential roles of these migrasomes (Fig. 4A). In line with our initial observations, during in vitro culture of cortical bone, emigrating cells promoted formation of structures resembling retraction fibers of migrasomes at their trailing edges (Fig. 4B). Over the course of OI, migrating OPSCs progressively secreted increasing numbers of migrasomes, which were wheat germ agglutinin (WGA)-positive, through retraction fibers surrounding these cells (Fig. 4C to E). Since tetraspanin 4 (TSPAN4) is highly expressed in migrasomes [28], we generated a stable TSPAN4-GFP expressing OPSC cell line to precisely delineate migrasomes dynamics during osteogenesis. Seven days after OI, we detected abundant calcium deposits co-localizing with both retraction fibers and freely released migrasomes (Fig. 4F). With the objective to further investigate the functional impact of these migrasomes, we employed an optimized protocol to completely abolish the cells while preserving the native deposited migrasomes (Fig. 4G). Remarkably, after enzymatic digestion, migrasomes remained firmly adhered to the surface (Fig. 4H), exhibiting a significantly higher abundance in the OI group compared to the control group (Fig. 4I). Next, to delineate the functional role of these residing migrasomes, OPSCs were cultured for 7 days under either OI (O7) or DMEM (D7), followed by complete cell removal (CR). The resulting substrates were either directly stained with ARS staining or reseeded with cells and cultured for additional 7 days in DMEM prior to ARS analysis (O7-D7 and D7-D7, respectively). Rewardingly, residual migrasomes derived from osteogenically induced OPSCs (O7-D7) potentiated both initiation and progression of ossification (Fig. 4J), as well as overexpression of osteogenic genes including *ALP*, *OCN*, *RUNX2*, and *BMP2* compared to O7-D7 samples (Fig. 4K to M)*.* Altogether, these results demonstrate that OI stimulates the production of migrasomes with a sustained capacity to orchestrate osteogenesis.

### Mitochondria–mineral–migrasome complex formation

**Fig. 5.**
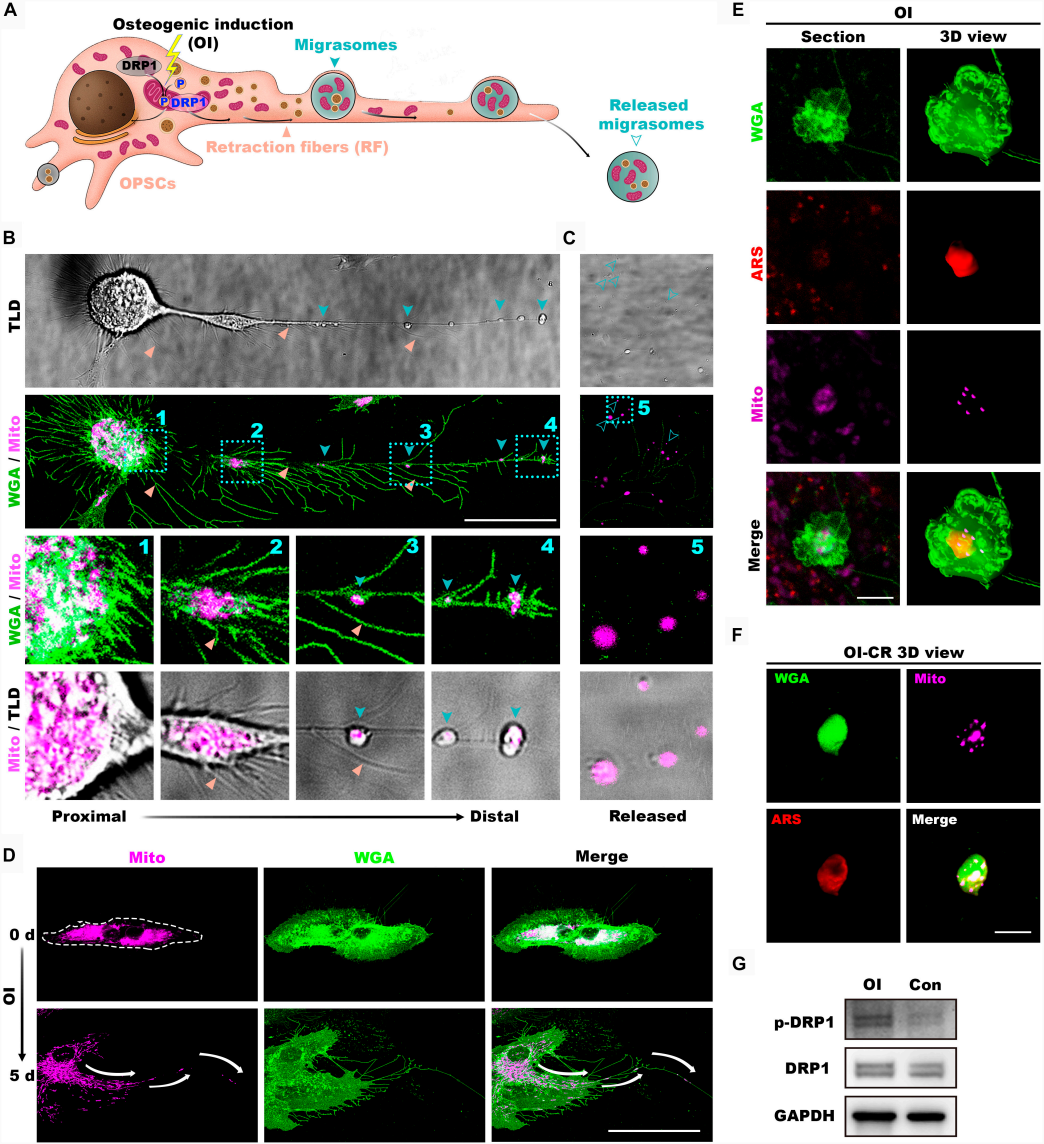
Osteogenic induction drives mitochondrial transport outward and the formation of the mitochondria–mineral–migrasome (MMM) complex. (A) Schematic illustration of OI-induced mitochondrial fission in cells and the composition of the MMM complex. (B) Live-cell fluorescence microphotographs depicting the distribution of mitochondria from the proximal to distal region of OPSCs (1, inside the cell; 2, in the cell tail; 3–4, in the migrasomes). (C) At distal sites, retraction fibers thinned and fragmented, releasing migrasome-encapsulated mitochondria. (D) Fluorescence microphotographs featuring mitochondria migrating peripherally and externalizing 0 and 5 days of OI. (E) Representative immunofluorescence microphotographs and 3D reconstruction revealing extracellular MMM triads. (F) Persistent co-localization of the MMM after CR. (G) Western blot analysis of total and phosphorylated mitochondrial fission-related proteins Drp1 after OI. Scale bars in µm: 100 (D), 30 (B), 5 (F), and 2 (E).

Previous studies have demonstrated that mitochondria can serve as carriers for amorphous calcium phosphate, transporting it extracellularly to initiate biomineralization [29]. Building upon our finding that OI stimulates production of migrasomes, we hypothesized that they may act as carriers of mitochondria to promote extracellular matrix mineralization (Fig. 5A). Live-cell fluorescence microscopy revealed that after 7 days of OI, mitochondria in OPSCs migrated toward the cell trailing edge and became enclosed within migrasomes connected by retraction fibers (Fig. 5B). Subsequent thinning and fragmentation of these fibers led to the release of migrasome-encapsulated mitochondria (Fig. 5C). Longitudinal fluorescence imaging consistently revealed relocation of mitochondria toward the peripheral cytoplasm and their subsequent externalization during OI (Fig. 5D). Strikingly, even after complete decellularization, migrasomes encapsulated both mitochondria and calcium deposits, forming a mitochondria–mineral–migrasome (MMM) complex (Fig. 5E and F). Mechanistically, both TSPAN4 overexpression and OI enhanced the levels of total and phosphorylated mitochondrial fission protein DRP1 (Fig. 5G). In conclusion, these findings indicate that OI activates DRP1-mediated mitochondrial fission and externalization via migrasomes, leading to the formation of the MMM complex as a fundamental unit of extracellular mineralization.

### Preparation of migrasome-functionalized BCP scaffolds

**Fig. 6.**
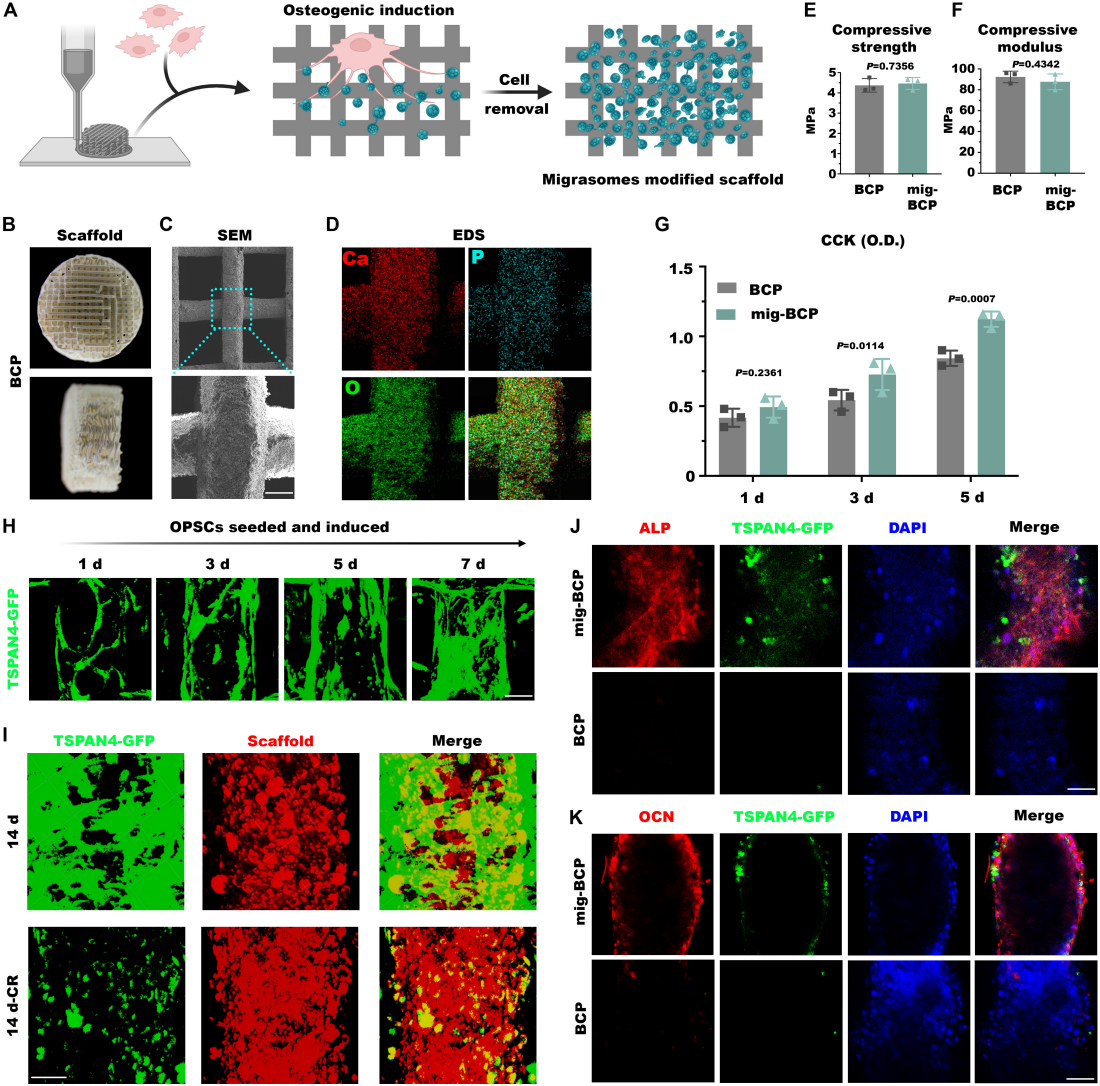
Fabrication of migrasome-functionalized BCP scaffolds. (A to D) Schematic diagram (A), macrophotographs of front and side views (B), SEM microphotographs (C), and EDS mapping demonstrating the distribution of Ca, P, and O elements (D) in migrasome-functionalized BCP (mig-BCP) scaffolds. (E and F) BCP scaffolds with or without migrasome-functionalization exhibited comparable compressive strength (E) and modulus (F). (G) CCK-8 assay revealed enhanced cell proliferation on mig-BCP scaffolds compared to the BCP control. (H) Confocal laser scanning microscopy 3D reconstruction of TSPAN4^+^ OPSCs on scaffolds at different OI time points. (I) The 3D reconstruction fluorescence images of scaffolds on day 14 of culture before and after decellularization. (J and K) Representative immunofluorescence microphotographs demonstrating more abundant expression of ALP (J) and OCN (K) in bone marrow mesenchymal stem cells (BMSCs) cultured on mig-BCP scaffolds compared to BCP controls. Scale bars: 20 μm.

With the objective to explore the translational potential of migrasomes for tissue engineering applications, we precultured OPSCs under OI on various biomaterial surfaces, followed by decellularization to obtain bioactive interfaces enriched with migrasomes. Rewardingly, migrasome successfully loaded on both titanium sheets, silk fibroin membranes, and BCP scaffolds (Fig. S9). Due to the 3D printability of the latter, we further investigated their potential as a carrier for migrasome delivery (Fig. 6A). Predominantly composed of O, P, and Ca, BCP scaffolds were fabricated into a cylindrical form featuring a columnar architecture with micro-rough surfaces (Fig. 6B to D). Importantly, functionalization did not compromise the mechanical properties, as the compressive strength and modulus remained unaltered between the unmodified BCP and the migrasome-functionalized BCP (mig-BCP) materials (Fig. 6E and F). Furthermore, OPSC effectively adhered and proliferated on BCP scaffolds, with significantly enhanced cell viability observed on mig-BCP surfaces (Fig. 6G). Next, we precultured TSPAN4^+^ OPSCs on the BCP scaffold. Upon prolonged culture and induction, the cells gradually up-regulated TSPAN4-GFP and progressively proliferated, extensively interconnecting with each other to cover nearly the whole surface by day 14 (Fig. S10 and Fig. 6H). Thereafter, numerous migrasomes retained on the scaffold even following decellularization (Fig. 6I and Fig. S11). To directly assess the osteoinductive capacity of these mig-BCP scaffolds, we seeded BMSCs onto the constructs. After 7 days of culture, the mig-BCP group scaffolds retained a large number of intact TSPAN4-GFP-positive migrasomes. Strikingly, BMSCs cultured on mig-BCP scaffolds markedly up-regulated osteogenic markers ALP and OCN (Fig. 6J and K).

### Mig-BCP scaffolds promote the regeneration of cranial bone defects in mice

**Fig. 7.**
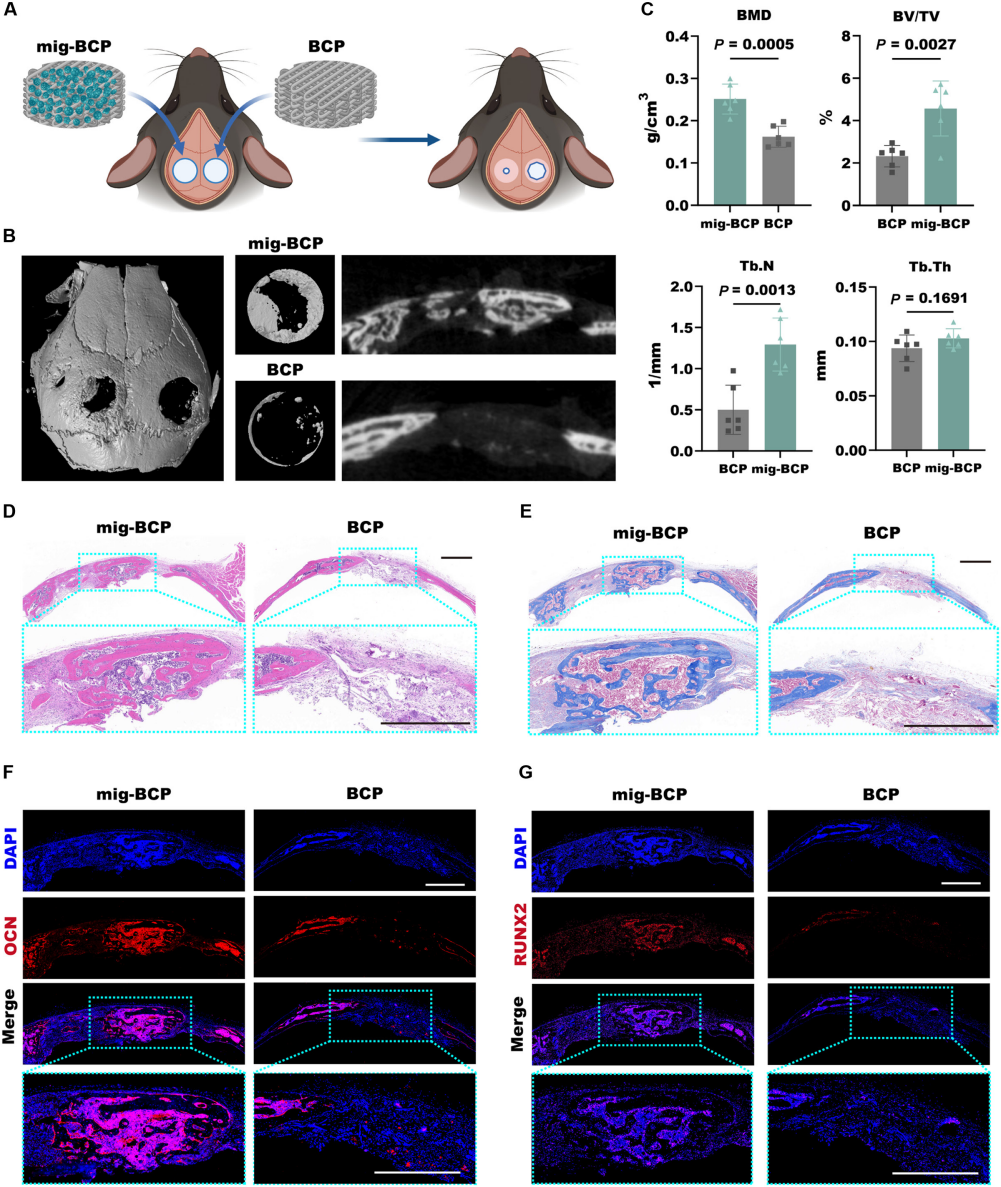
Migrasome-functionalized BCP scaffolds promote cranial bone defect regeneration in mice. (A to C) Schematic illustration (A) micro-computed tomography reconstructions (B) and bone morphometry analysis (C) of mouse cranial defect regeneration experiment 8 weeks after surgery revealing substantial new bone formation, elevated bone mineral density (BMD), percent bone volume (BV/TV), and trabecular number (Tb.N), but not trabecular thickness (Tb.Th), in defects treated with mig-BCP scaffolds compared to limited regeneration in BCP controls (n=6). (D and E) Representative microphotographs H&E (D) and Masson's trichrome (E) staining revealing extensive central bone tissue with vascularization and collagen deposition (blue) within new bone matrix of mig-BCP defects, as opposed to peripheral bone thickening in BCP controls. (F and G) Representative immunofluorescence microphotographs demonstrating upregulated OCN (F) and RUNX2 (G) expression in the mig-BCP group. Scale bars: 1 mm.

To validate the therapeutic efficacy of mig-BCP scaffolds in vivo, we implanted them into a critical-sized murine calvarial bone defect model, utilizing unmodified BCP scaffolds as a control (Fig. 7A). Eight weeks after surgery, mig-BCP scaffold significantly enhanced new bone formation, leading to a reduction in the defect area, in contrast to the BCP cohort, which demonstrated minimal osteogenesis confined to the defect margins (Fig. 7B). While trabecular thickness remained comparable in both groups, mig-BCP-treated animals revealed significantly higher bone density, volume percentage, and trabecular number (Fig. 7C). Consistently, the central region of the regenerating bone defect in the mig-BCP-treated animals revealed extensive vascular infiltration and new bone formation, in sharp contrast to the marginal thickening and scarce vascularization in the BCP implants (Fig. 7D and E). Finally, whereas expression of the osteogenic markers OCN and RUNX2 were confined to the periphery of the defect in the controls, it was markedly elevated in the newly formed bone tissue of the mig-BCP group (Fig. 7F and G). Altogether, these data confirm that migrasome-functionalized interfaces successfully promote vascularized bone regeneration, establishing a highly effective cell-free strategy for bone defect repair.

## Discussion

Repairing large segmental bone defects requires sustained osteoinductive signaling at the defect site. While cell-free tissue engineering, particularly strategies utilizing biomaterials and exosomes, has emerged as a promising therapeutic alternative [30,31]. Here, we propose a highly effective bone regeneration strategy wherein OPSCs are cultured on scaffold surfaces under OI, while their resulting migrasome-enriched ECM is used to functionalize the scaffold. We demonstrate that this approach enhances osteogenesis and establishes a biomimetic platform that redefines the application of migrasomes in regenerative materials science and positions the native, migrasome-functionalized ECM as a novel bioactive interface for cell-free bone repair.

To endow biomaterials with osteoinductive signals, conventional cell-based therapies typically involve the direct implantation of osteogenic cells or stem cells, which to a certain extent can provide sustained secretion of bioactive factors and matrix remodeling. However, these approaches suffer from low cell survival, unpredictable cell fate, immune and safety risks, and complex regulatory requirements [32]. In contrast, growth factor-based therapies, exemplified by BMP2, represent one of the few FDA-approved osteoinductive strategies [33] capable of promoting bone formation, but often requiring supraphysiological doses to maintain effective signaling. Furthermore, the high cost, short half-life, and associated side effects, such as ectopic ossification and inflammatory responses, restrict the practical application of such growth factor-based approaches [34]. With the objective to overcome these transplantation-associated barriers, extracellular vesicle therapies, particularly employing exosomes, have been recognized for their ability to partially mimic cellular paracrine functions by delivering multiple endogenous signaling molecules to synergistically regulate osteogenesis. However, exosome-based therapies still face practical challenges, including complex preparation procedures, low purification efficiency, poor batch-to-batch consistency, and unstable integration with scaffold materials, which impede their potential for repairing large-volume bone defects [35]. Consequently, there is an urgent need for a cell-free signaling platform that provides sustained endogenous osteoinductive capability while stably integrating with scaffold surfaces. In line with this goal, migrasomes, a newly discovered class of cell-derived structures, offer new possibilities for bone regeneration. Unlike conventional extracellular vesicles, migrasomes are formed during cell migration and are naturally enriched in and adherent to the ECM [10]. Their formation is tightly coupled with cellular behavior and the microenvironment. This unique feature allows migrasomes to serve not only as carriers of signaling molecules but also as a spatially stable and functionally sustained bioactive interface.

Cell-free therapeutics typically harness bioactive components derived from diverse cellular sources to achieve different therapeutic purposes [36]. To obtain bioactive components with potent osteogenic capacity, we focused on OPSCs, a population of cells derived from cortical bone with inherently high osteogenic potential. While culturing the bone fragments, we demonstrated that these cells actively migrate outward through the lacunar–canalicular network (LCN), which functions as a conduit for osteocyte dendrites in the bone matrix [37]. Together with the lacunae housing osteocyte cell bodies, their dendrites form an intricate architectural system. Moreover, the LCN likely serves as a mechanosensory apparatus enabling environmental sensing and potential cellular translocation of osteocytes, which are known to exhibit micromotion within their lacunae [38]. In this work, we confirmed that the migrating cells possess a robust osteogenic mineralization capacity. Indeed, upon OI, our dual-crosslinked hydrogel/OPSC constructs underwent gradual mineralization and contraction, progressively forming 3D bone-like organoids. Following 4 weeks of induction, these organoids developed lamellar Col 1 matrices structurally analogous to native bone. Remarkably, this mineralization dynamics phenocopies the developmental pattern of the jawbone, when stem cells directly differentiate into osteoblasts, secreting extracellular matrices wherein they embed themselves, ultimately establishing hierarchical bone deposition [39].

Given the robust osteogenic and mineralization capacities of OPSCs, we selected these cells as the parental source to further investigate the therapeutic utility of their derived migrasomes. When using standard isolation methods, we demonstrated that in contrast to uninduced control, OI strongly promotes anchoring of migrasomes produced by OPSCs to the bottom of the culture dish. Strikingly, upon reseeding OPSCs onto the culture dish, we confirmed that the migrasomes deposited during OI exert a pronounced pro-osteogenic effect. These findings inspired a strategy to endow scaffold materials with a bioactive surface interface by leveraging the migrasome-enriched ECM produced by OI-stimulated OPSCs. By implementing an optimized decellularization procedure, we successfully deposited this migrasome-enriched ECM onto BCP scaffolds, thereby conferring the scaffold materials with excellent osteoinductive properties. This bioactive surface engineering strategy conceptualizes cells as “coating workers”: by modulating cellular states through conditioned culture, cells are induced to generate the desired ECM in situ. Altogether, this approach departs from the conventional view of cells as raw materials that require subsequent purification and enrichment of their components [40].

During osteogenic mineralization, mitochondria may transport amorphous calcium phosphate to the extracellular space, leading to the formation of calcified nodule deposits [41]. Here, we suggested that during OI, phosphorylation of the mitochondrial fission-related protein DRP1 may promote the gradual translocation of mitochondria toward the extracellular space, where they colocalize with migrasomes and mineralized nodules, thus forming the MMM complexes. Interestingly, exposure of mitochondria to mild stress, such as carbonyl cyanide 3-chlorophenylhydrazone, leads to the export of damaged mitochondria extracellularly via the migrasome-dependent pathway [15], supporting our data that OI may similarly act as a mild stimulant, activating mitochondrial fission and externalization. However, the precise role of mitochondria in linking osteogenic mineralization with migrasome dynamics remains to be further elucidated.

In conclusion, our study demonstrates that OI effectively promotes OPSCs to generate abundant migrasome-enriched ECM with pro-osteogenic activity. This matrix can naturally form an in situ bioactive surface interface, thereby endowing scaffold materials with strong osteoinductive properties. This inspired us to explore a transformative cell-free therapeutics paradigm. It shifts from conventional methodologies requiring both vesicle harvesting and artificial material conjugation to a pioneering strategy involving direct cell preculture on biomaterial surfaces, thus facilitating migrasome deposition, with subsequent decellularization promoting intrinsic vesicle-matrix integration. Altogether, this platform delineates a versatile and highly effective solution to simplify the application of migrasomes and enhance their binding efficiency, opening a new frontier for their application in regenerative medicine.

## Materials and Methods

### Isolation and culture of OPSCs

Human cortical bone fragments were harvested from the buccal and occlusal tissues removed during the extraction of impacted mandibular third molars at Shanghai Ninth People’s Hospital, Shanghai Jiao Tong University School of Medicine, as approved by its Ethics Committee (approval no. SH9H-2020-T36-2).

Murine cortical bone fragments were obtained from the mandible of 8-week-old mice.

Following the isolation of human or murine cortical bone fragments, periosteal and marrow tissues were removed via phosphate-buffered saline (PBS) irrigation and mechanical curettage. The cortical bone was rinsed with sterile PBS and cut into small fragments. The fragments were evenly distributed onto the bottom of culture dishes supplemented with complete culture medium.

Cell proliferation was assessed using a 5-ethynyl-2′-deoxyuridine (EdU) incorporation assay (Beyotime, ST067) following the manufacturer’s instructions. Briefly, after counterstaining the nuclei with 4′,6-diamidino-2-phenylindole (DAPI), EdU-positive cells were visualized using a fluorescence microscope. The proliferation rate was calculated as the percentage of EdU-positive cells relative to the total number of DAPI-stained nuclei.

Cell viability was evaluated using a Cell Counting Kit-8 (Dojindo, Japan) according to the manufacturer’s instructions measuring the absorbance at 450 nm using a microplate reader. Cell viability was expressed as the optical density (OD) value.

### Lentiviral transduction and stable cell lines construction

To generate stable TSPAN4-GFP- or GFP-expressing cell lines, we utilized the respective lentiviral vectors (Genomeditech, Shanghai, China) following the standard procedures for efficient viral infection and gene expression in OPSCs. Briefly, the lentiviral particles were added to the cell culture medium for 12 h. Next, cells were selected in fresh medium containing 5 μg/ml puromycin until drug-resistant colonies became visible. After 72 h, we confirmed successful GFP expression and assessed the efficiency of transfection and expression via fluorescence microscopy.

### Trilineage differentiation induction and detection

To assess the differentiation potential, we conducted trilineage induction assays.

For OI, cells were cultured in OI medium consisting of DMEM supplemented with 10% fetal bovine serum, 40 μg/ml ascorbic acid (Sigma-Aldrich, A4544), 2 mg/ml β-glycerophosphate (Sigma-Aldrich, G9422), and 100 nM dexamethasone (Sigma-Aldrich, D4902). Following induction, cells were rinsed with PBS and fixed in 4% paraformaldehyde. The cells were then rinsed with distilled water and stained with ARS solution (Beyotime, C0140).

For chondrogenic induction, cells were cultured in chondrogenic induction medium containing DMEM, 50 μg/ml ascorbic acid, 10 nM dexamethasone, and 10 ng/ml TGF-β3 (PeproTech, 100-36E).

For adipogenic induction, cells were cultured and stained according to the manufacturer’s instructions (Cyagen, HUXMA-90031).

### Preparation of dual-crosslinked hydrogel and bone organoids

To establish a biomimetic 3D OPSCs culture system, we prepared dual-crosslinked hydrogels. Briefly, alginate methacryloyl (Engineering for life, EFL-AlgMA) was dissolved in sterile deionized water at a concentration of 2% (w/v), followed by supplementation with 0.5% (w/v) of the photoinitiator LAP to facilitate light-induced crosslinking. The hydrogel solution was thoroughly mixed, preincubated for 15 min to ensure homogeneity, and utilized to resuspend OPSCs. Next, the cell-laden hydrogel was transferred into a transwell plate followed by photoinitiator-assisted photopolymerization via 30 s exposure to 365-nm UV light to induce initial chemical crosslinking. Subsequently, the hydrogel was cultured in OI medium, which facilitated secondary ionic crosslinking through the calcium salts generated by the osteogenic differentiation of the cells.

### Decellularization

To isolate native migrasomes on biomaterial surfaces, cells were cultured on dishes or scaffolds with or without OI. Next, the constructs were decellularized using an optimized protocol to completely abolish all cellular components while preserving the deposited migrasomes. Briefly, the cells were washed 3 times with PBS and subjected to a mild decellularization process in 0.25% trypsin-EDTA at 37 °C for 30 min, followed by gentle mechanical dissociation via pipetting. To effectively eliminate residual cells within the scaffolds, they were extensively washed by gentle pipetting from all directions.

### Fabrication and characterization of 3D-printed BCP scaffolds

To precisely control the generation of the scaffolds, BCP powder (HA/β-TCP = 20/80 wt%, particle size 1 to 5 μm, Sigma-Aldrich) was mixed with a 15 wt% polyvinyl alcohol binder solution at a solid loading of 45 vol% to form a homogeneous paste. The slurry was loaded into a syringe-based 3D bioprinter equipped with a nozzle. Scaffolds with orthogonal pore architecture were printed at an extrusion pressure of 250 kPa and a printing speed of 15 mm/s, dried at 60 °C for 24 h, followed by debinding at 600 °C (2 °C/min), sintering at 1,150 °C for 3 h (heating rate 5 °C/min), and autoclaving prior to experimental use.

For mechanical characterization, BCP scaffolds were subjected to uniaxial compression testing using a universal testing machine. Each scaffold was compressed between 2 parallel plates at a constant crosshead speed until failure, while force and displacement were recorded in real time. The compressive strength was calculated as the maximum load divided by the original cross-sectional area, and the elastic modulus was derived from the slope of the linear elastic region of the stress–strain curve.

### Animal experiments

DMP1-cre, GFP-Tg, tdTomato, WT C57BL/6J mice, and BALB/c nude mice of both sexes were used and crossbred to generate the transgenic lines utilized in this study. The breeding and experiments were approved by the Ethics Committee of Shanghai Ninth People’s Hospital, Shanghai Jiao Tong University School of Medicine (approval no. SH9H-2019-A665-1).

For the early in vivo osteogenic differentiation experiment, OPSCs were precultured in OI medium supplemented with 50 ng/ml BMP2 for 7 days. Next, OPSCs were encapsulated in a gelatin sponge and bilaterally implanted into dorsal subcutaneous pockets of 8-week-old male BALB/c nude mice (*n* = 6) anesthetized with isoflurane, and small incisions were made bilaterally in the dorsal subcutaneous region. After 2 weeks, the implants from the euthanized mice were harvested for histological analysis.

For in vivo chondrogenic differentiation experiments, silk fibroin scaffolds with or without OPSCs preloaded with TGF-β3 were similarly implanted subcutaneously to 8-week-old male BALB/c nude mice (*n* = 6). To evaluate cartilage-like tissue formation, the implants were harvested for histological analyses after 4 weeks.

To establish a clinically relevant in vivo model of critical-sized bone trauma, calvarial defects (4 mm diameter) were generated in 10-week-old male C57BL/6 mice (*n* = 6). Under anesthesia via isoflurane inhalation and continuous saline irrigation, a midline scalp incision and periosteal elevation were performed, followed by creation of bilateral defects in the parietal bones 2 mm posterior to the bregma using a trephine bur. Experimental and control groups received 3D-printed scaffolds with or without migrasomes, respectively. After suturing the skin, buprenorphine was administered every 12 h for 48 h postsurgery. Defect regeneration was analyzed 8 weeks after surgery by micro-computed tomography (CT) and histomorphometry. After decalcification, the bone tissue was sectioned and stained for histological analyses.

### Histological analyses and imaging

Cortical bone fragments harvested from transgenic mice ware cultured in a dish followed by 3D confocal microscopy.

To selectively evaluate the expression of key cellular markers, immunofluorescence and flow cytometry were performed according to standard protocols on a confocal fluorescence microscope (Leica TCS SP8, Germany) or a flow cytometer (BD LSRFortessa X-20 cell analyzer, USA), respectively, using the following immunofluorescence antibodies: CD29, CD90, CD105, CD45 (mesenchymal stromal cell marker, Abcam, ab93758), STRO-1 (Abcam, AB214086), CD34 (Abcam, ab81289), and CD73 (Abcam, ab317462); and flow cytometer antibodies: CD29 (BD, 561794), CD90 (BD, 561972), CD105 (BD, 560839), STRO-1 (Santa Cruz, sc-47733), CD34 (BD, 561440), CD45 (BD, 560915), and CD73 (BD, 561014) in concentrations recommended by the manufacturer.

Osteogenic and chondrogenic differentiation assays were conducted according to the previously established protocol to assess the differentiation potential. Sections and cells were stained with anti-SOX-9 (Abcam, ab185966) and anti-Col 2 (DSHB, II-II6B3) antibodies, followed by Alexa Fluor-conjugated secondary antibodies (Invitrogen) and DAPI (Sigma-Aldrich, D9542) for nucleus visualization. For live cell ARS staining, 0.5 μg/ml ARS was added into medium for 3 days (Beyotime, ST1078).

To detect cell proliferation, DiO staining was performed using a DiO cell membrane stain (Thermo Fisher Scientific, V22886) according to the manufacturer’s instructions. To track migrasome and mitochondria dynamics, cells were incubated with Alexa Fluor 488-conjugated WGA (Thermo Fisher Scientific, W11261) and MitoBright (DOJINDO, Japan, MT12). The dish was placed on the Nanolive stage (3D cell explorer-Fluo, Nanolive, Switzerland) for live cell imaging.

For histological analyses, harvested samples were immediately rinsed with PBS and fixed in 4% paraformaldehyde at 4 °C for 24 h. Following fixation, the specimens were cryoprotected by sequential immersion in 15% and 30% sucrose solutions at 4 °C until complete infiltration. The tissues were then embedded in optimal cutting temperature compound and rapidly frozen. For paraffin embedding, after decalcification with 20% EDTA, the tissues were dehydrated, embedded in paraffin, and sectioned. Hematoxylin and eosin staining, Masson’s staining, and immunofluorescence staining were performed according to standard protocols. The antibodies used were as follows: RUNX2 (Abcam, ab192256) and OCN (Abcam, ab309521).

### Scanning electron microscopy

The samples were fixed in 2.5% glutaraldehyde at 4 °C for 2 h, rinsed 3 times with PBS, and dehydrated through a graded ethanol series (30%, 50%, 70%, 90%, and 100%) for 15 min per step followed by critical point-drying. After coating with a thin layer of gold-palladium alloy, the samples were observed under a scanning electron microscope (Phenom Scientific, Netherlands) at an acceleration voltage of 1 to 5 kV. For mineralized areas, energy-dispersive x-ray spectroscopy was used to analyze the elemental composition of the samples.

### Quantitative real-time PCR

Total RNA was isolated from the aforementioned cells using TRIzol reagent (Invitrogen, USA) according to the manufacturer’s instructions, followed by RNA concentration and purity detection by a NanoDrop spectrophotometer (Allsheng, China). Total RNA (1,000 ng) was reverse transcribed into complementary DNA (cDNA) using Hifair II 1st Strand cDNA Synthesis SuperMix (Yeasen Biotechnology, China). Quantitative real-time polymerase chain reaction (qRT-PCR) was performed using a Roche LightCycler 480 II (Roche, Switzerland). The initial denaturation step was performed for 10 min at 95 °C, followed by 40 cycles of 95 °C for 10 s and 60 °C for 60 s.

### Western blot

Cells were harvested and lysed in radioimmunoprecipitation assay buffer (Sigma-Aldrich, R0278), supplemented with a protease inhibitor cocktail (Thermo Fisher Scientific, A32959). The protein concentration of the lysates was determined using a BCA Protein Assay Kit (Sigma-Aldrich, 71285) according to the manufacturer’s instructions. Western blotting was performed according to standard protocols with the following antibodies: BMP2 (Abcam, ab284387), Col 2 (Abcam, ab307674), SOX-9 (Abcam, ab185966), p-DRP1 (Abcam, ab314755), DRP1 (Abcam, ab184247), and GAPDH (Abcam, ab8245) followed by signal detection by a gel imaging system (UVItec, UK).

### Micro-CT analysis

The samples were scanned using a micro-CT system (SkyScan 1176; Bruker micro-CT, Kontich, Belgium) with the following parameters: source voltage, 65 kV; source current, 280 μA; Al 1.0-mm filter; pixel size, 18 μm; and rotation step, 0.55°. The section through the center of the implant, oriented in the sagittal and coronal planes, was used as the measurement section. CTAn (Bruker micro-CT, Kontich, Belgium) was used to calculate bone mineral density (BMD), percent bone volume (BV/TV), trabecular thickness (Tb.Th), and trabecular number (Tb.N).

### Statistical analysis

Data are presented as mean ± standard deviation (SD). Statistical analyses were performed using GraphPad Prism (v10.1). Normality was assessed using the Shapiro–Wilk test. For normally distributed data with homogeneous variance, Student *t* test or one-way analysis of variance followed by Tukey’s post hoc test were applied. Nonnormally distributed or heteroscedastic data were analyzed using the Kruskal–Wallis test.

## Data Availability

All data are available in the main text or the Supplementary Materials.

## References

[1] Kim M, Wang X, Li Y, Lin Z, Collins CP, Liu Y, Ahn Y, Tsal H-M, Song JW, Duan C, et al. Personalized composite scaffolds for accelerated cell- and growth factor-free craniofacial bone regeneration. Bioact Mater. 2024;41:427–439. 39188380 10.1016/j.bioactmat.2024.07.029 PMC11345904

[2] Tai A, Landao-Bassonga E, Chen Z, Tran M, Allan B, Ruan R, Calder D, Goonewardene M, Ngo H, Zheng MH. Systematic evaluation of three porcine-derived collagen membranes for guided bone regeneration. Biomater Transl. 2023;4(1):41–50. 37206304 10.12336/biomatertransl.2023.01.006 PMC10189808

[3] Jepsen S, Schwarz F, Cordaro L, Derks J, Hämmerle CHF, Heitz-Mayfield LJ, Hernández-Alfaro F, Meijer HJA, Naenni N, Ortiz-Vigón A, et al. Regeneration of alveolar ridge defects. Consensus report of group 4 of the 15th European workshop on periodontology on bone regeneration. J Clin Periodontol. 2019;46(Suppl 21):277–286. 31038223 10.1111/jcpe.13121

[4] Miron RJ. Optimized bone grafting. Periodontol 2000. 2024;94(1):143–160. 37610202 10.1111/prd.12517

[5] Xu X, Song J. Segmental long bone regeneration guided by degradable synthetic polymeric scaffolds. Biomater Transl. 2020;1(1):33–45. 35837653 10.3877/cma.j.issn.2096-112X.2020.01.004 PMC9255814

[6] Yang C, Xue Y, Duan Y, Mao C, Wan M. Extracellular vesicles and their engineering strategies, delivery systems, and biomedical applications. J Control Release. 2024;365:1089–1123. 38065416 10.1016/j.jconrel.2023.11.057

[7] Jin J. Stem cell treatments. JAMA. 2017;317(3):330. 28114555 10.1001/jama.2016.17822

[8] Freeman FE, Pitacco P, van Dommelen, Nulty J, Browe DC, Shin J-Y, Alsberg E, Kelly DJ. 3D bioprinting spatiotemporally defined patterns of growth factors to tightly control tissue regeneration. Sci Adv. 2020;6(33):eabb5093. 32851179 10.1126/sciadv.abb5093 PMC7428335

[9] Wu Y, Mao J, Zhou Y, Hong G, Wu H, Hu Z, Huang X, Shi J, Xie Z, Lan Y. Youthful brain-derived extracellular vesicle-loaded GelMA hydrogel promotes scarless wound healing in aged skin by modulating senescence and mitochondrial function. Research. 2025;8:0644. 40161249 10.34133/research.0644 PMC11951976

[10] Wu D, Xu Y, Ding T, Zu Y, Yang C, Yu L. Pairing of integrins with ECM proteins determines migrasome formation. Cell Res. 2017;27(11):1397–1400. 28829047 10.1038/cr.2017.108 PMC5674152

[11] Jiao H, Yu L. Migrasomes: Biogenesis, physiological roles, and therapeutic potentials. J Cell Biol. 2024;223(11): Article e202403051. 39400310 10.1083/jcb.202403051 PMC11473597

[12] Pan S, Yin Z, Shi C, Xiu H, Wu G, Heng Y, Zhu Z, Zhang J, Gui J, Yu Z, et al. Multifunctional injectable hydrogel microparticles loaded with miR-29a abundant BMSCs derived exosomes enhanced bone regeneration by regulating osteogenesis and angiogenesis. Small. 2024;20(16): Article e2306721. 38018340 10.1002/smll.202306721

[13] Han L, Liu H, Fu H, Hu Y, Fang W, Liu J. Exosome-delivered BMP-2 and polyaspartic acid promotes tendon bone healing in rotator cuff tear via Smad/RUNX2 signaling pathway. Bioengineered. 2022;13(1):1459–1475. 35258414 10.1080/21655979.2021.2019871 PMC8805918

[14] D’Souza A, Burch A, Dave KM, Sreeram A, Reynolds MJ, Dobbins DX, Kamte YS, Zhao W, Sabatelle C, Joy GM, et al. Microvesicles transfer mitochondria and increase mitochondrial function in brain endothelial cells. J Control Release. 2021;338:505–526. 10.1016/j.jconrel.2021.08.038 PMC8526414 34450196

[15] Jiao H, Jiang D, Hu X, Du W, Ji L, Yang Y, Li X, Sho T, Wang X, Li Y, et al. Mitocytosis, a migrasome-mediated mitochondrial quality-control process. Cell. 2021;184(11):2896–2910.e13. 34048705 10.1016/j.cell.2021.04.027

[16] Zhang F, Liu W, Mao Y, Yang Y, Ling C, Liu Y, Yao F, Zhen Y, Wang X, Zou M. Migrasome, a migration-dependent organelle. Front Cell Dev Biol. 2024;12:1417242. 38903534 10.3389/fcell.2024.1417242 PMC11187097

[17] Wang M, Zhang S, Kutibiding A, Zhou X, Tian X, Yuan S, Lv M, Yang Y, Bai S. M2 macrophage-derived migrasomes enhance bone regeneration by directing osteogenic differentiation of bone marrow mesenchymal stem cells (BMSCs). Tissue Cell. 2026;100: Article 103394. 41713111 10.1016/j.tice.2026.103394

[18] Zhang S, Wang M, Kutibiding A, Liu D, Manafu T, Zhao W, Yang Y. Dynamic crosstalk between Tspan4^+^ macrophage subsets and MSCs via migrasomes orchestrates fracture repair. Front Cell Dev Biol. 2025;13:1666465. 41584843 10.3389/fcell.2025.1666465 PMC12827626

[19] Jiang D, Jiao L, Li Q, Xie R, Jia H, Wang SH, Chen Y, Liu S, Huang D, Zheng J, et al. Neutrophil-derived migrasomes are an essential part of the coagulation system. Nat Cell Biol. 2024;26(7):1110–1123. 38997457 10.1038/s41556-024-01440-9 PMC11251984

[20] Zhang C, Li T, Yin S, Gao M, He H, Li Y, Jiang D, Shi M, Wang J, Yu L. Monocytes deposit migrasomes to promote embryonic angiogenesis. Nat Cell Biol. 2022;24(12):1726–1738. 36443426 10.1038/s41556-022-01026-3

[21] Hu L, Ma L, Li H, Lin W, Wang L, Lu M, Shen H, Liu S, Jian J, Qiu L, et al. Macrophage lineage cells-derived migrasomes activate complement-dependent blood-brain barrier damage in cerebral amyloid angiopathy mouse model. Nat Commun. 2023;14(1):3945. 37402721 10.1038/s41467-023-39693-x PMC10319857

[22] Zhang Y, Bi J, Huang J, Tang Y, Du S, Li P. Exosome: A review of its classification, isolation techniques, storage, diagnostic and targeted therapy applications. Int J Nanomedicine. 2020;15:6917–6934. 33061359 10.2147/IJN.S264498 PMC7519827

[23] Li G, Zhao Y, Wang H, Zhang Y, Cai D, Zhang Y, Song W. The M2 macrophages derived migrasomes from the surface of titania nanotubes array as a new concept for enhancing osteogenesis. Adv Healthc Mater. 2024;13(20): Article e2400257. 38520188 10.1002/adhm.202400257

[24] Zhang X, Yao L, Meng Y, Li B, Yang Y, Gao F. Migrasome: A new functional extracellular vesicle. Cell Death Discov. 2023;9(1):381. 37852963 10.1038/s41420-023-01673-x PMC10584828

[25] Wang S, Zhao J, Zhang W, Ye D, Zhang X, Zou D, Zhang X, Sun X, Sun S, Zhang W, et al. Comprehensive evaluation of cryopreserved bone-derived osteoblasts for the repair of segmental mandibular defects in canines. Clin Implant Dent Relat Res. 2015;17(4):798–810. 24131659 10.1111/cid.12164

[26] Wang S, Zhang W, Zhao J, Ye D, Zhu C, Yang Y, Zhang X, Sun X, Yang C, Jiang X, et al. Long-term outcome of cryopreserved bone-derived osteoblasts for bone regeneration in vivo. Biomaterials. 2011;32(20):4546–4555. 21459433 10.1016/j.biomaterials.2011.03.014

[27] Dzamukova M, Brunner TM, Miotla-Zarebska J, Heinrich F, Brylka L, Mashreghi M-F, Kusumbe A, Kühn R, Schinke T, Vincent TL, et al. Mechanical forces couple bone matrix mineralization with inhibition of angiogenesis to limit adolescent bone growth. Nat Commun. 2022;13(1):3059. 35650194 10.1038/s41467-022-30618-8 PMC9160028

[28] Huang Y, Zucker B, Zhang S, Elias S, Zhu Y, Chen H, Ding T, Li Y, Sun Y, Lou J, et al. Migrasome formation is mediated by assembly of micron-scale tetraspanin macrodomains. Nat Cell Biol. 2019;21(8):991–1002. 31371828 10.1038/s41556-019-0367-5

[29] Ling Z, Ge X, Jin C, Song Z, Zhang H, Fu Y, Zheng K, Xu R, Jiang H. Copper doped bioactive glass promotes matrix vesicles-mediated biomineralization via osteoblast mitophagy and mitochondrial dynamics during bone regeneration. Bioact Mater. 2025;46:195–212. 39760064 10.1016/j.bioactmat.2024.12.010 PMC11699476

[30] Yu T, Zhao IS, Pan H, Yang J, Wang H, Deng Y, Zhang Y. Extracellular vesicle-functionalized bioactive scaffolds for bone regeneration. Asian J Pharm Sci. 2024;19(5): Article 100945. 10.1016/j.ajps.2024.100945 PMC11525715 39483718

[31] Wang K-N, Li Z-Z, Zhou K, Liu B, Rao L, Bu L-L. Cell membrane-coated nanoparticles for dental, oral, and craniofacial diseases. Research. 2024;7:0478. 39296987 10.34133/research.0478 PMC11409001

[32] Zhang S, Xie D, Zhang Q. Mesenchymal stem cells plus bone repair materials as a therapeutic strategy for abnormal bone metabolism: Evidence of clinical efficacy and mechanisms of action implied. Pharmacol Res. 2021;172: Article 105851. 34450314 10.1016/j.phrs.2021.105851

[33] Hankenson KD, Gagne K, Shaughnessy M. Extracellular signaling molecules to promote fracture healing and bone regeneration. Adv Drug Deliv Rev. 2015;94:3–12. 26428617 10.1016/j.addr.2015.09.008

[34] Panos JA, Coenen MJ, Nagelli CV, McGlinch EB, Atasoy-Zeybek A, Lopez De Padilla C, Coghlan RF, Johnstone B, Ferreira E, Porter RM, et al. IL-1Ra gene transfer potentiates BMP2-mediated bone healing by redirecting osteogenesis toward endochondral ossification. Mol Ther. 2023;31(2):420–434. 36245128 10.1016/j.ymthe.2022.10.007 PMC9931547

[35] Dayanandan AP, Bello AB, Arai Y, Lee SJ, Lee S-H. Therapeutic strategy for exosome-based bone regeneration to osteoporosis: Challenges and potential solutions. J Adv Res. 2025; 10.1016/j.jare.2025.09.042.10.1016/j.jare.2025.09.04241015179

[36] Zhang L, Luo Z, Chen H, Wu X, Zhao Y. Glycyrrhizic acid hydrogel microparticles encapsulated with mesenchymal stem cell exosomes for wound healing. Research. 2024;7:0496. 39403257 10.34133/research.0496 PMC11471873

[37] Chang B, Liu X. Osteon: Structure, turnover, and regeneration. Tissue Eng Part B Rev. 2022;28(2):261–278. 33487116 10.1089/ten.teb.2020.0322 PMC9063188

[38] van Hove RP, Nolte PA, Vatsa A, Semeins CM, Salmon PL, Smit TH, Klein-Nulend J. Osteocyte morphology in human tibiae of different bone pathologies with different bone mineral density—Is there a role for mechanosensing? Bone. 2009;45(2):321–329. 19398046 10.1016/j.bone.2009.04.238

[39] Motoike S, Inada Y, Toguchida J, Kajiya M, Ikeya M. Jawbone-like organoids generated from human pluripotent stem cells. Nat Biomed Eng. 2025;9:1816–1834. 40603745 10.1038/s41551-025-01419-3 PMC12623242

[40] Kimiz-Gebologlu I, Oncel SS. Exosomes: Large-scale production, isolation, drug loading efficiency, and biodistribution and uptake. J Control Release. 2022;347:533–543. 35597405 10.1016/j.jconrel.2022.05.027

[41] Pei D-D, Sun J-L, Zhu C-H, Tian F-C, Jiao K, Anderson MR, Yiu C, Huang C, Jin C-X, Bergeron BE, et al. Contribution of mitophagy to cell-mediated mineralization: Revisiting a 50-year-old conundrum. Adv Sci. 2018;5(10):1800873. 10.1002/advs.201800873 PMC6193168 30356983

