# Supplementary material for: A Native Bioactive Interface Functionalized with Osteoprogenitor Stem Cell-Derived Migrasomes for Enhanced Bone Regeneration
Source: Research (Wash D C). 2026 Mar 30;9:1220. doi: 10.34133/research.1220 (PMC13033832; doi:10.34133/research.1220)
Supplement: Supplementary 1 — Figs. S1 to S11 [file research.1220.f1.zip › SUPPLEMENTARY MATERIALS.docx]

SUPPLEMENTARY MATERIALS

Figures S1 to S11


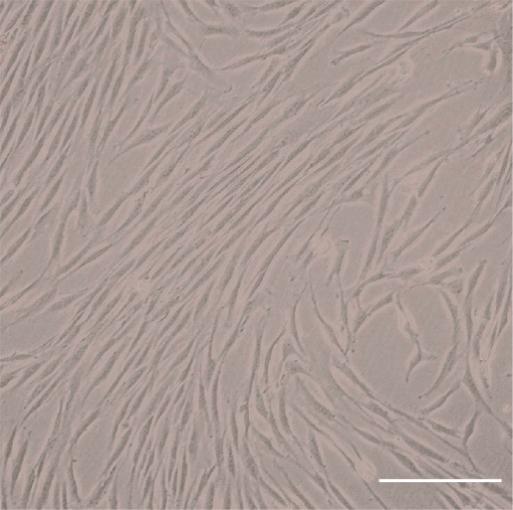


Fig. S1. OPSCs distal to the tissue exhibited an elongated spindle-shaped morphology and were arranged in whorled or bundled patterns. Scale bar: 50μm.


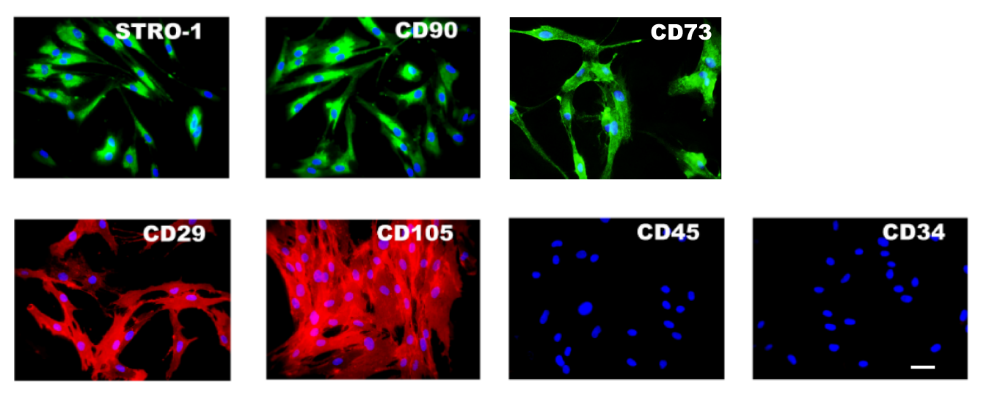


Fig. S2. Immunofluorescence staining revealed high expression of STRO-1, CD29, CD90, CD73, CD105, and low expression of CD34 and CD45 in OPSCs. Scale bar: 20μm.


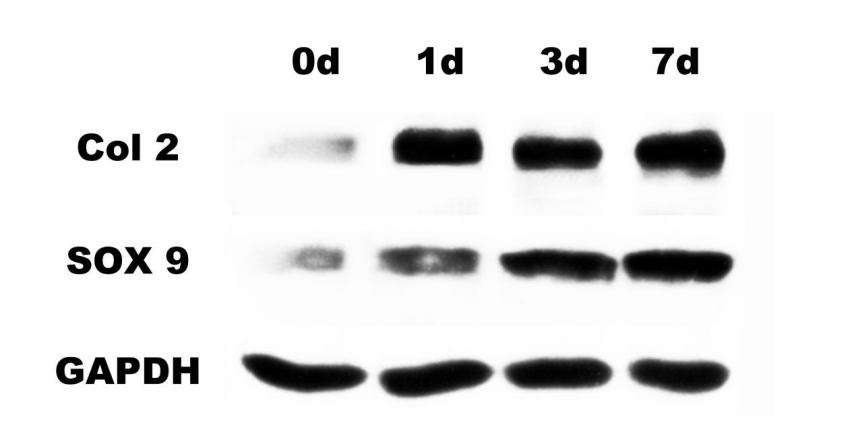


Fig. S3. Western blot showed after chondrogenic induction for 0, 1, 3 and 7 days, OPSCs exhibited upregulation expression of Col 2 and SOX-9 expression.


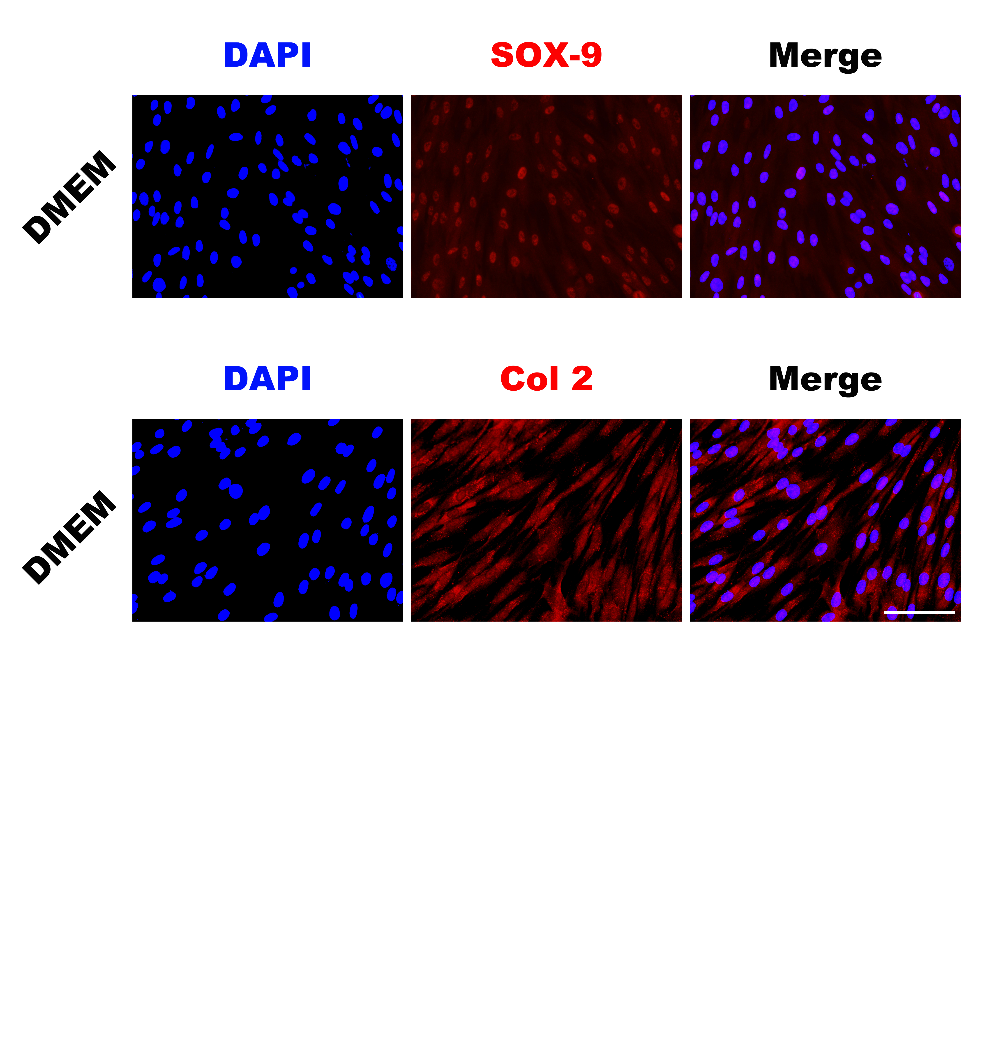


Fig. S4. Immunofluorescence staining showed the expressions of SOX-9 and Col-2 under DMEM culture conditions. Scale bar: 50μm.


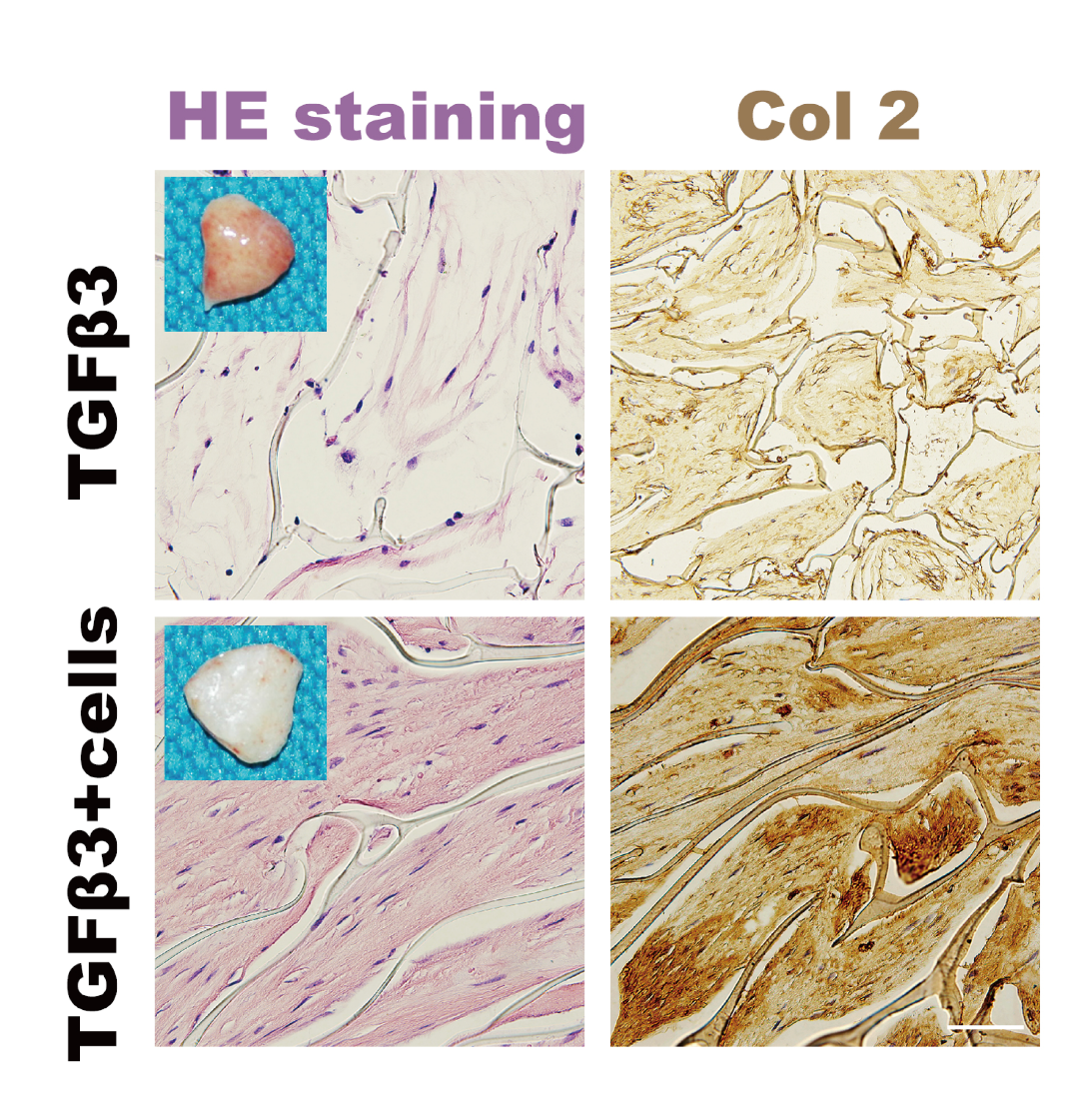


Fig. S5. Histological results demonstrated well-formed cartilage tissue with less vascular structures and more deposition of Col-2 fibers in the TGF-β3 + cells group. Scale bar: 100μm.


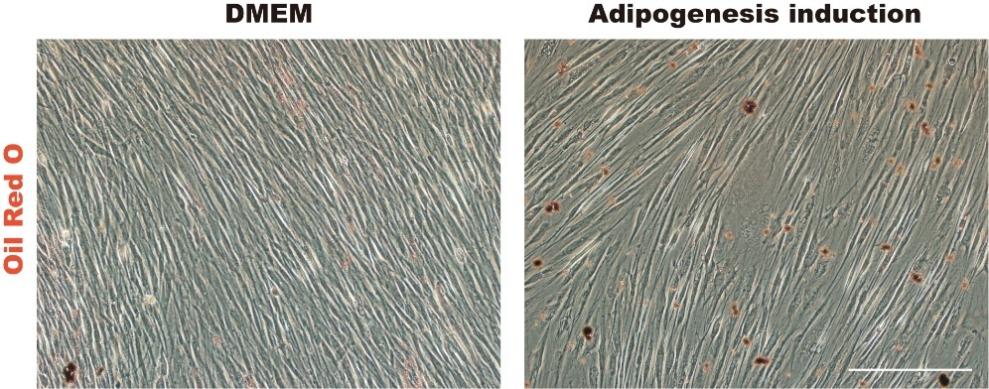


Fig. S6. Oil Red O staining revealed minimal lipid droplet formation after 21 days of induction. Scale bar: 100μm.


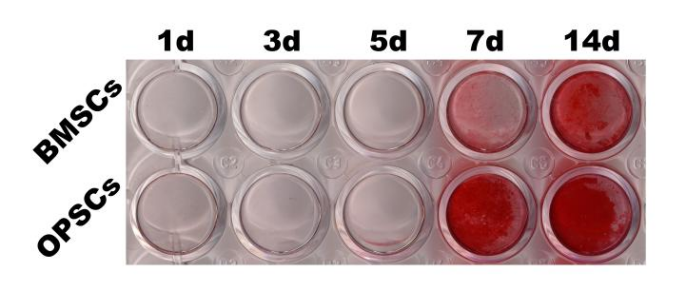


Fig. S7. The ARS staining revealed that OPSCs exhibited more calcium deposition compared to BMSCs.


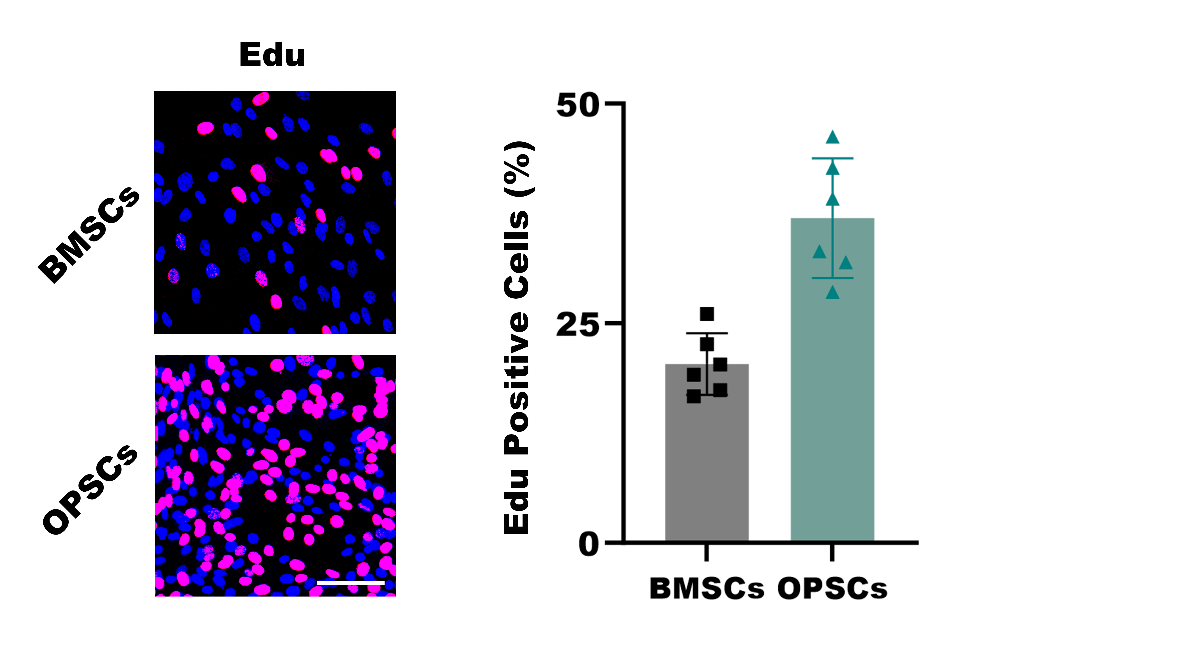


Fig. S8. The EdU assay revealed that OPSCs exhibited enhanced proliferative capacity compared with BMSCs. Scale bar: 50μm.


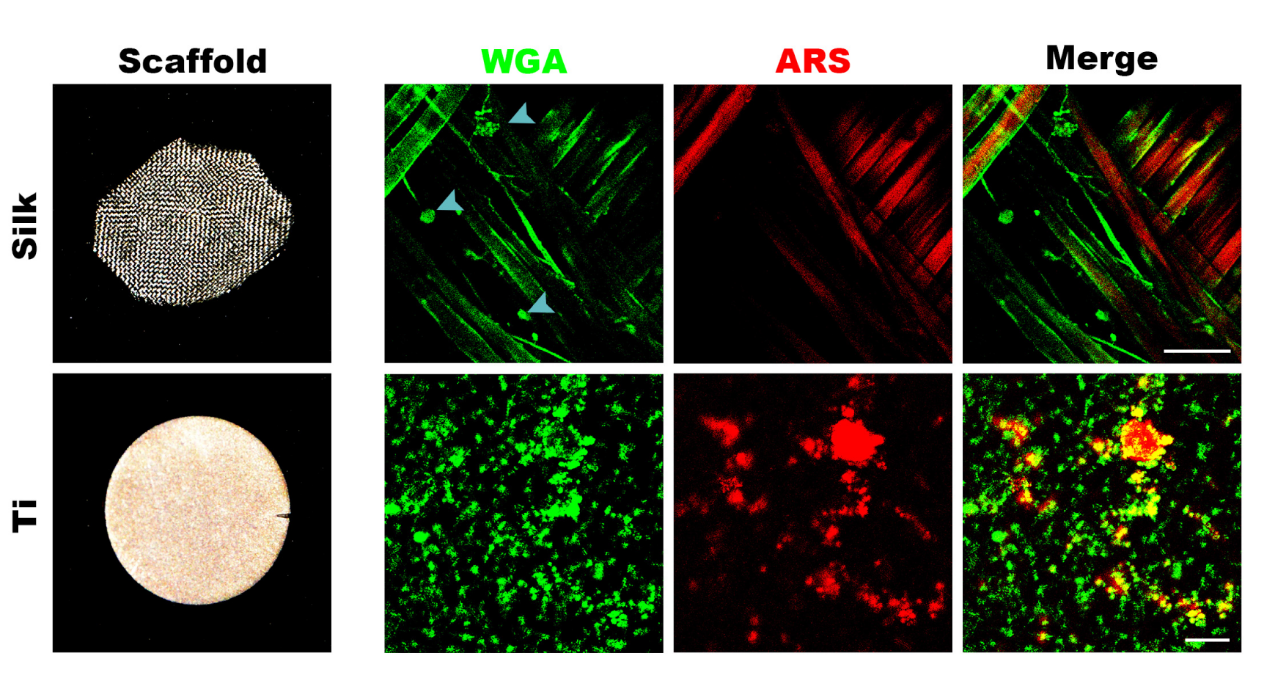


Fig. S9. Migrasome-modified silk fibroin electrospun membranes and titanium sheets: immunofluorescence images. Scale bar: 5μm.


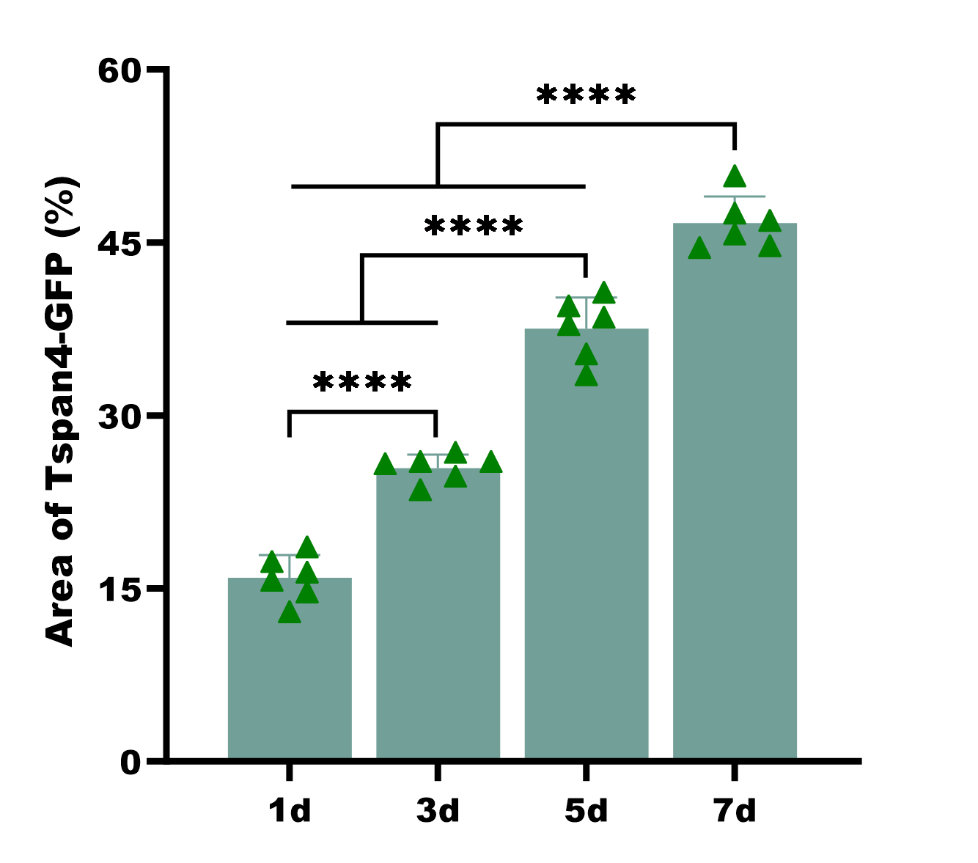


Fig. S10. Fluorescence quantification of Tspan4-GFP in OPSCs cultured on BCP scaffolds for the indicated time points.


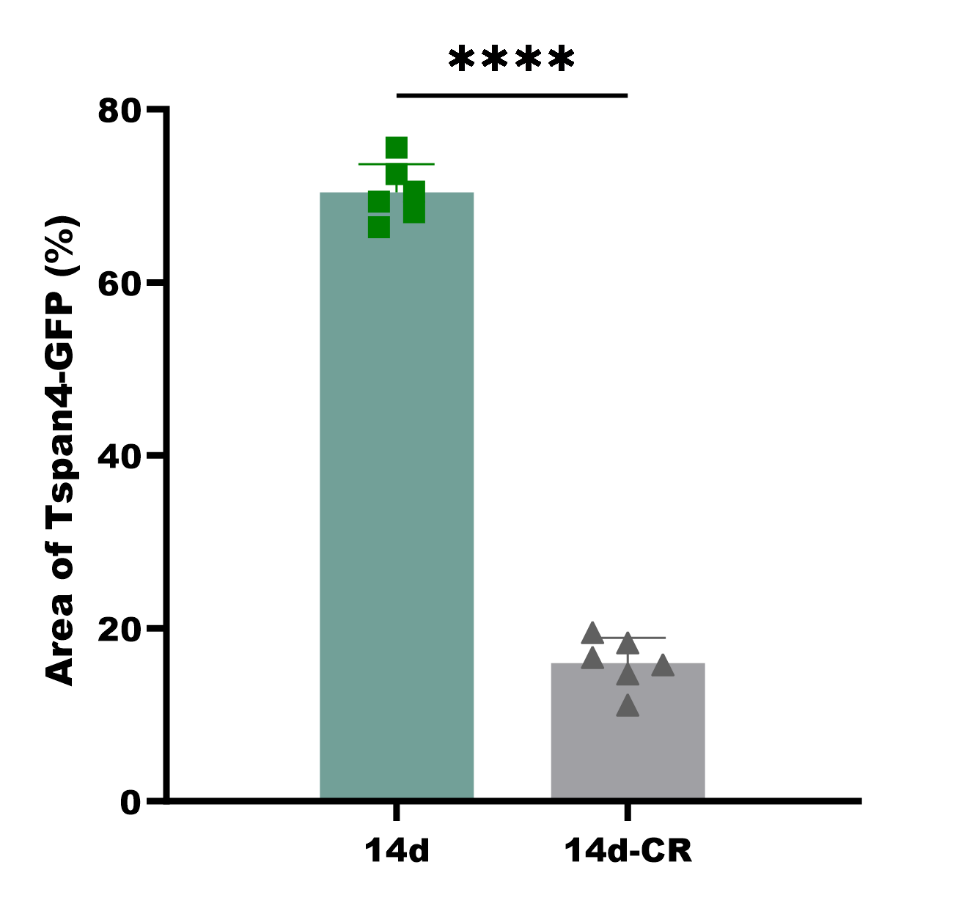


Fig. S11. Fluorescence quantification of Tspan4-GFP in OPSCs cultured on BCP scaffolds for 14 days, before and after CR.
