# Supplementary figures and images for: A Native Bioactive Interface Functionalized with Osteoprogenitor Stem Cell-Derived Migrasomes for Enhanced Bone Regeneration
Source: Research (Wash D C). 2026 Mar 30;9:1220. doi: 10.34133/research.1220 (PMC13033832; doi:10.34133/research.1220)

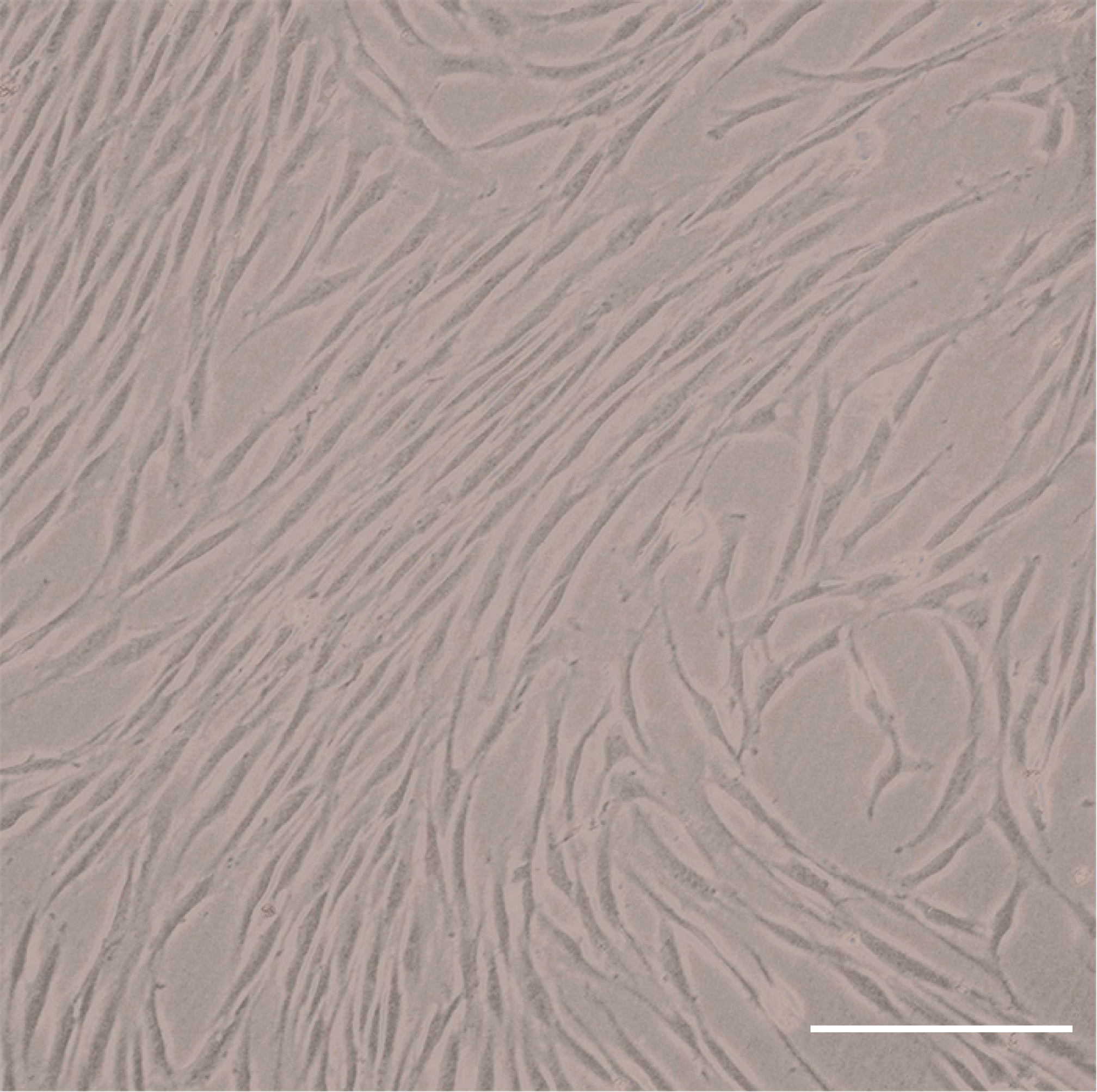

Supplement: Supplementary 1 — Figs. S1 to S11 [file research.1220.f1.zip › Figure s1.tif]

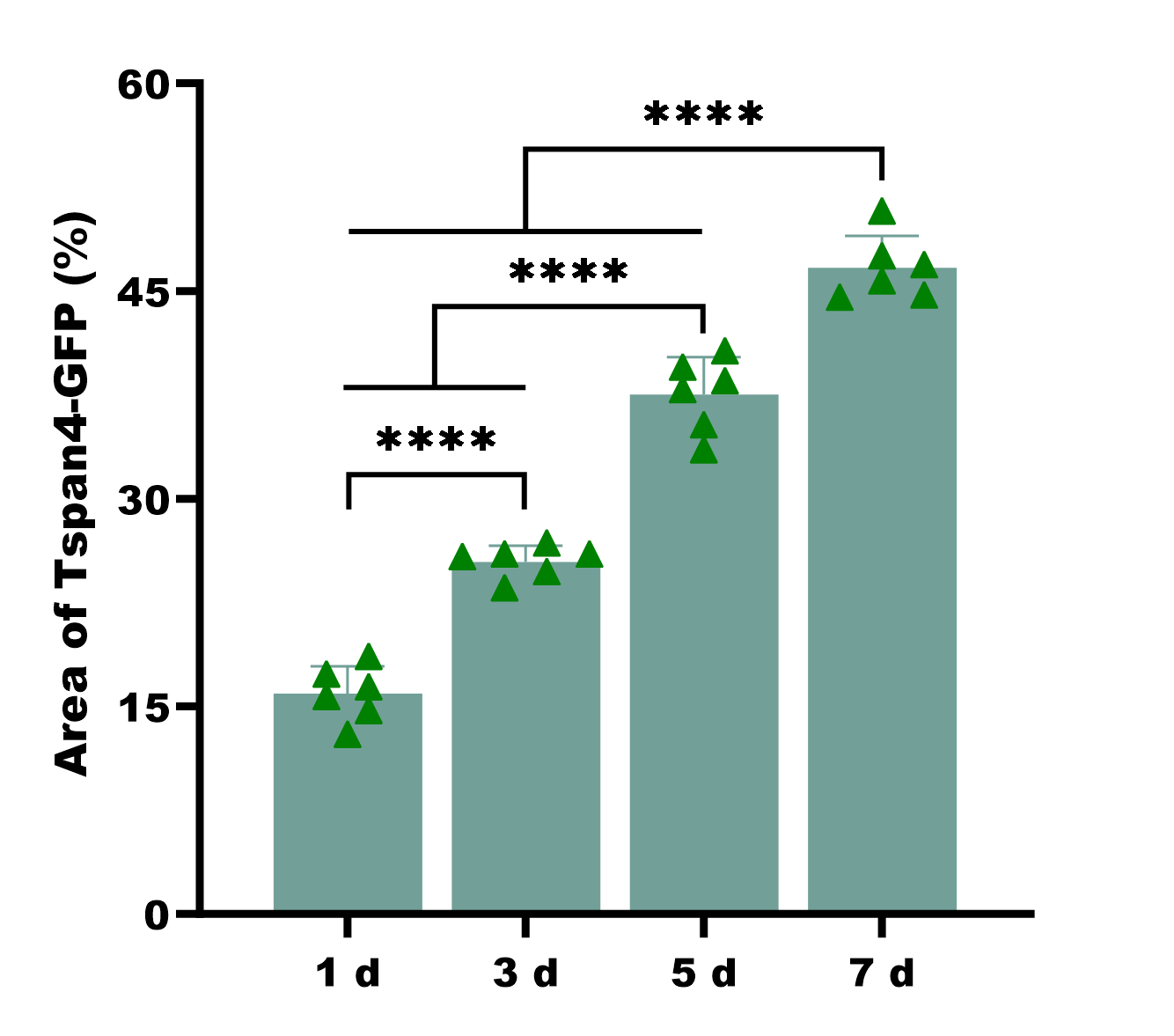

Supplement: Supplementary 1 — Figs. S1 to S11 [file research.1220.f1.zip › Figure S10.tif]

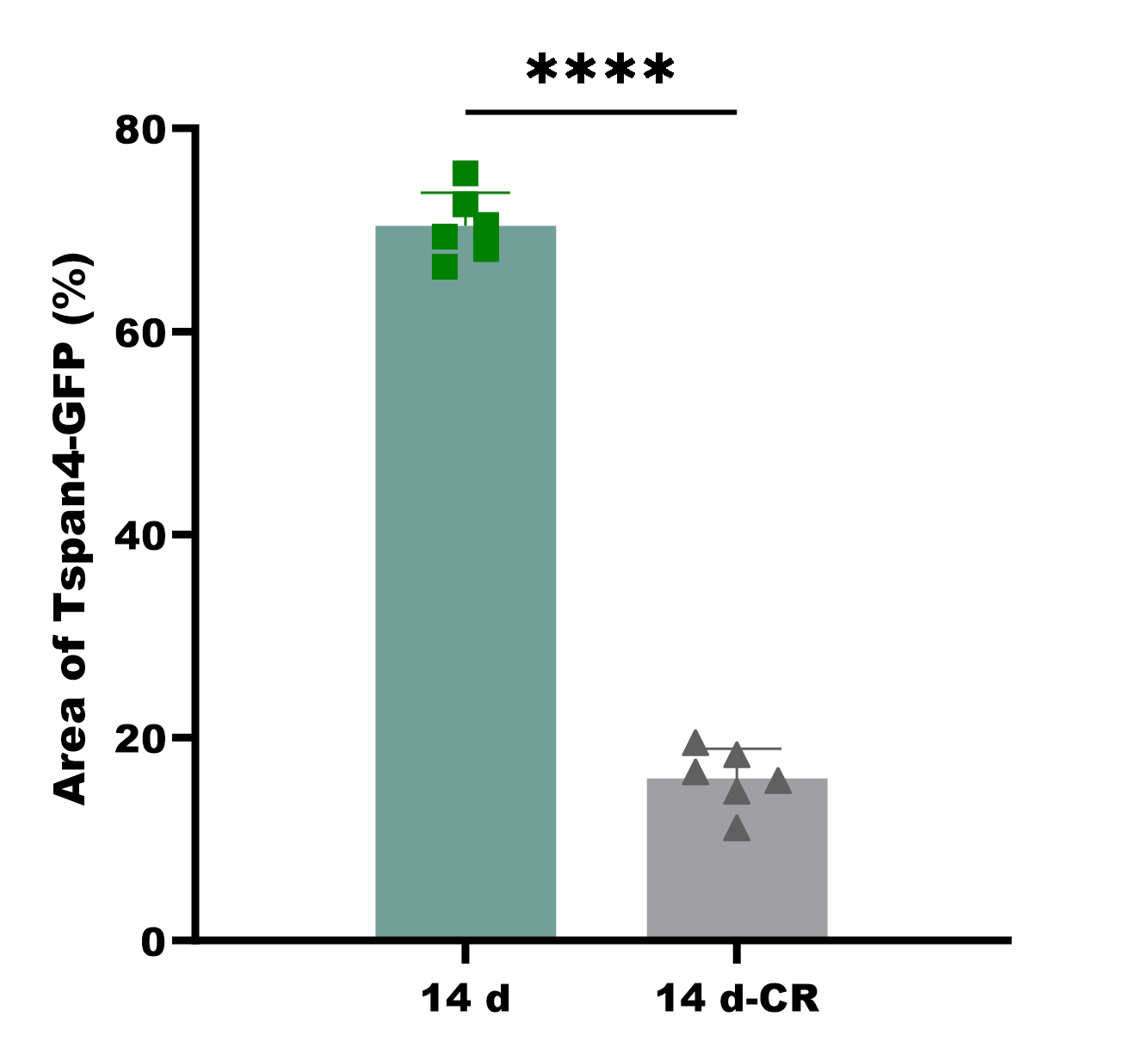

Supplement: Supplementary 1 — Figs. S1 to S11 [file research.1220.f1.zip › Figure S11.tif]

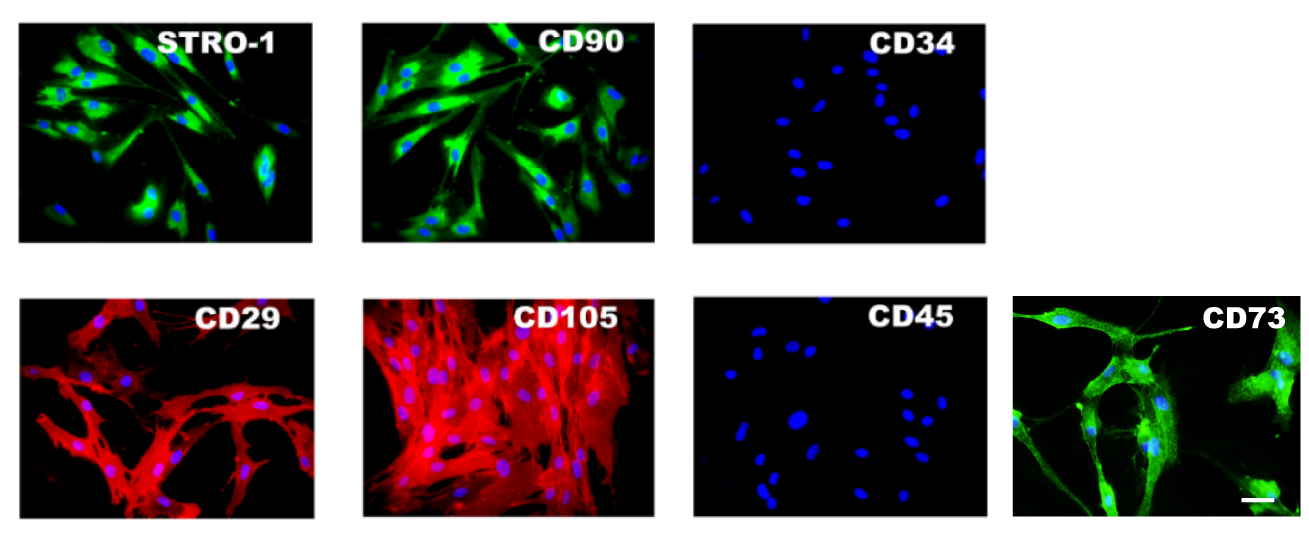

Supplement: Supplementary 1 — Figs. S1 to S11 [file research.1220.f1.zip › Figure s2.tif]

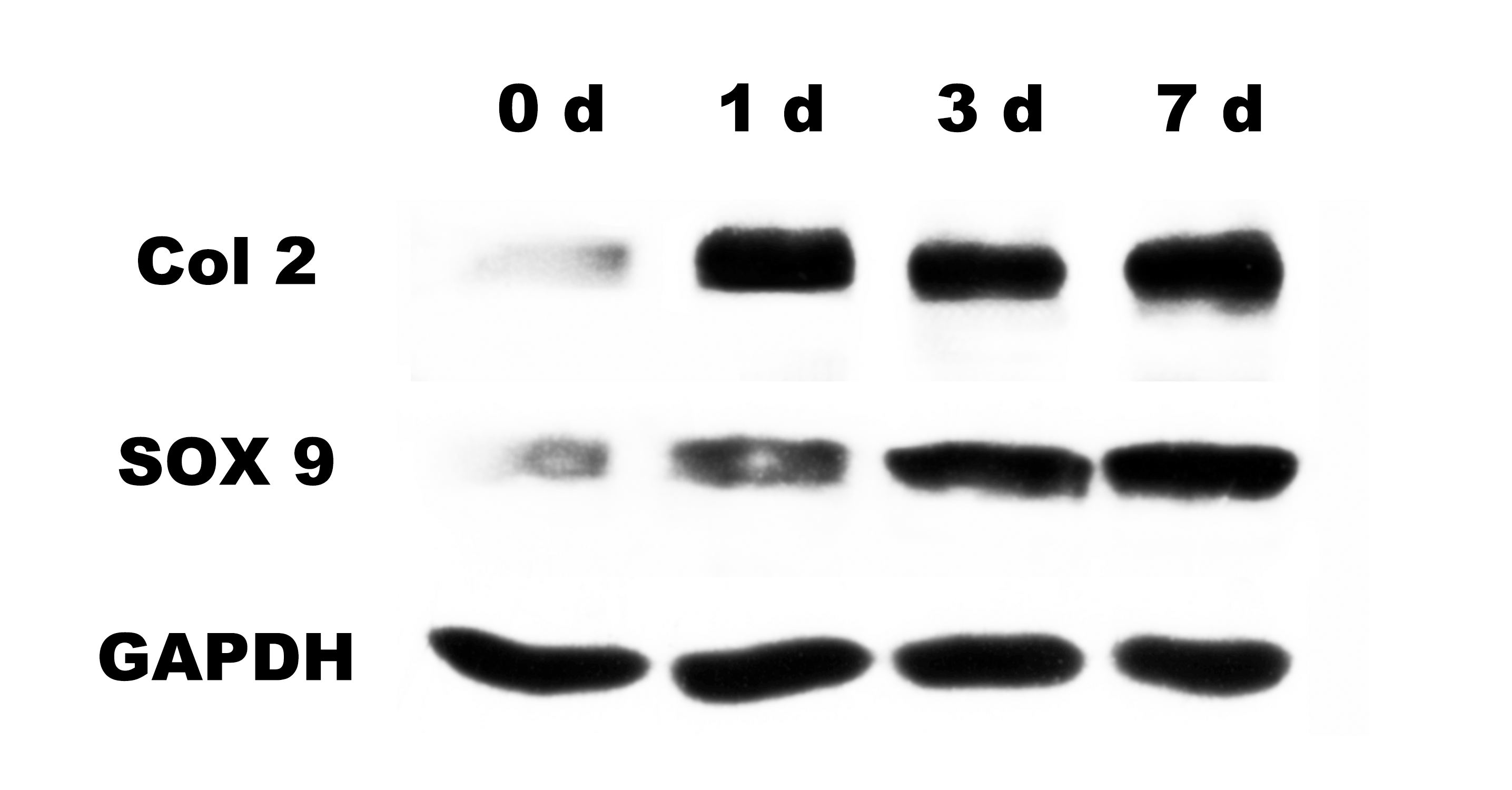

Supplement: Supplementary 1 — Figs. S1 to S11 [file research.1220.f1.zip › Figure s3.tif]

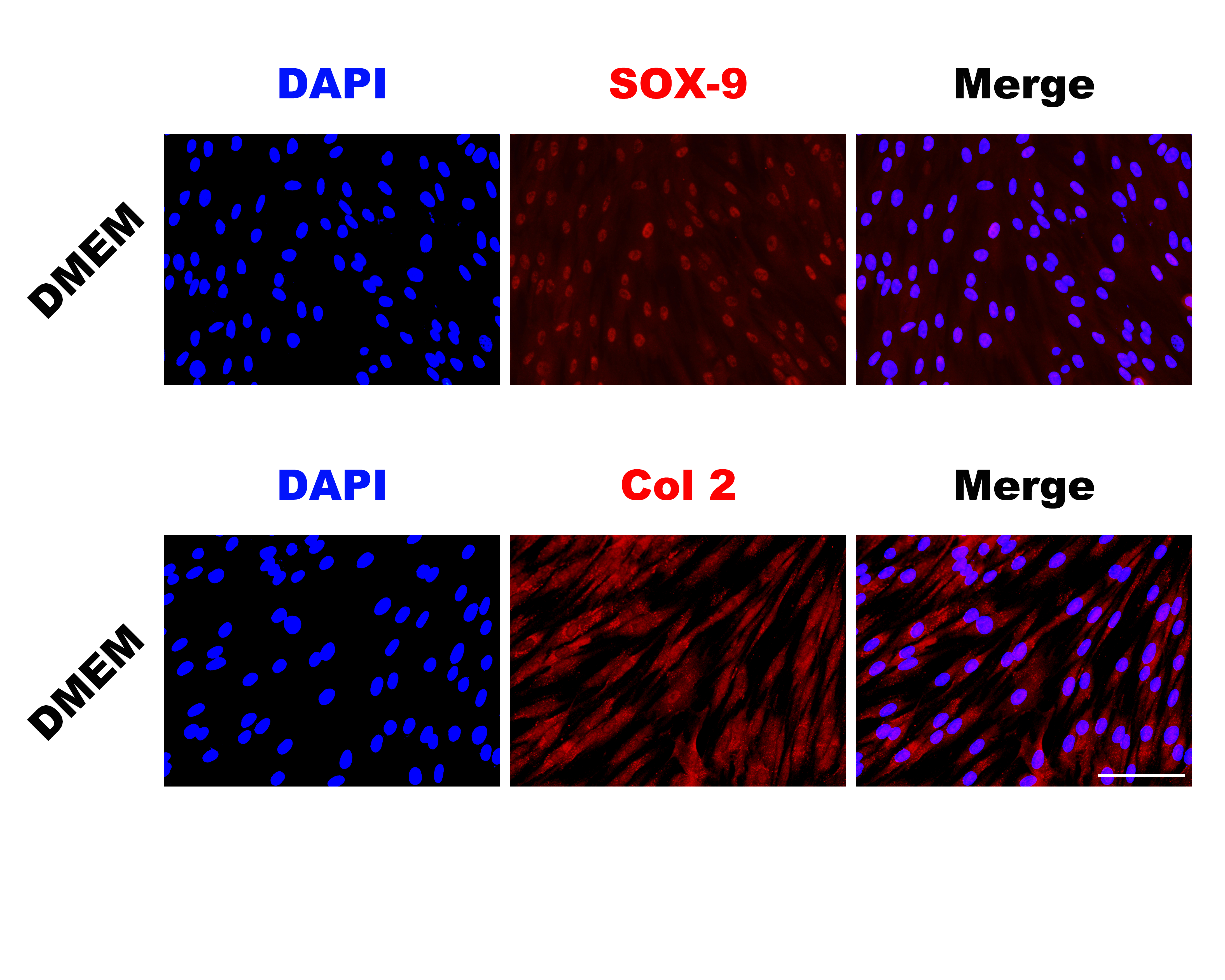

Supplement: Supplementary 1 — Figs. S1 to S11 [file research.1220.f1.zip › Figure s4.tif]

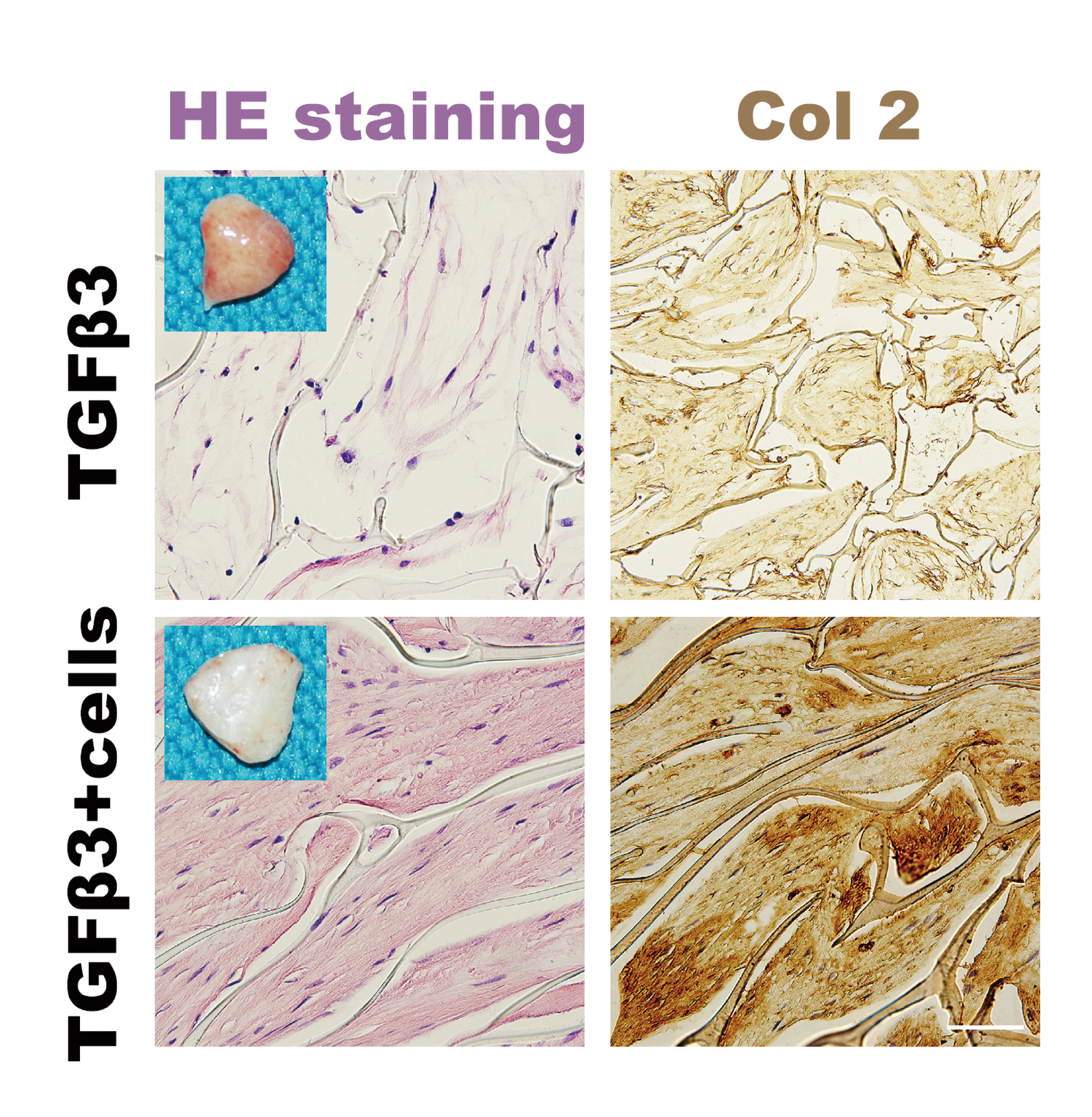

Supplement: Supplementary 1 — Figs. S1 to S11 [file research.1220.f1.zip › Figure S5.tif]

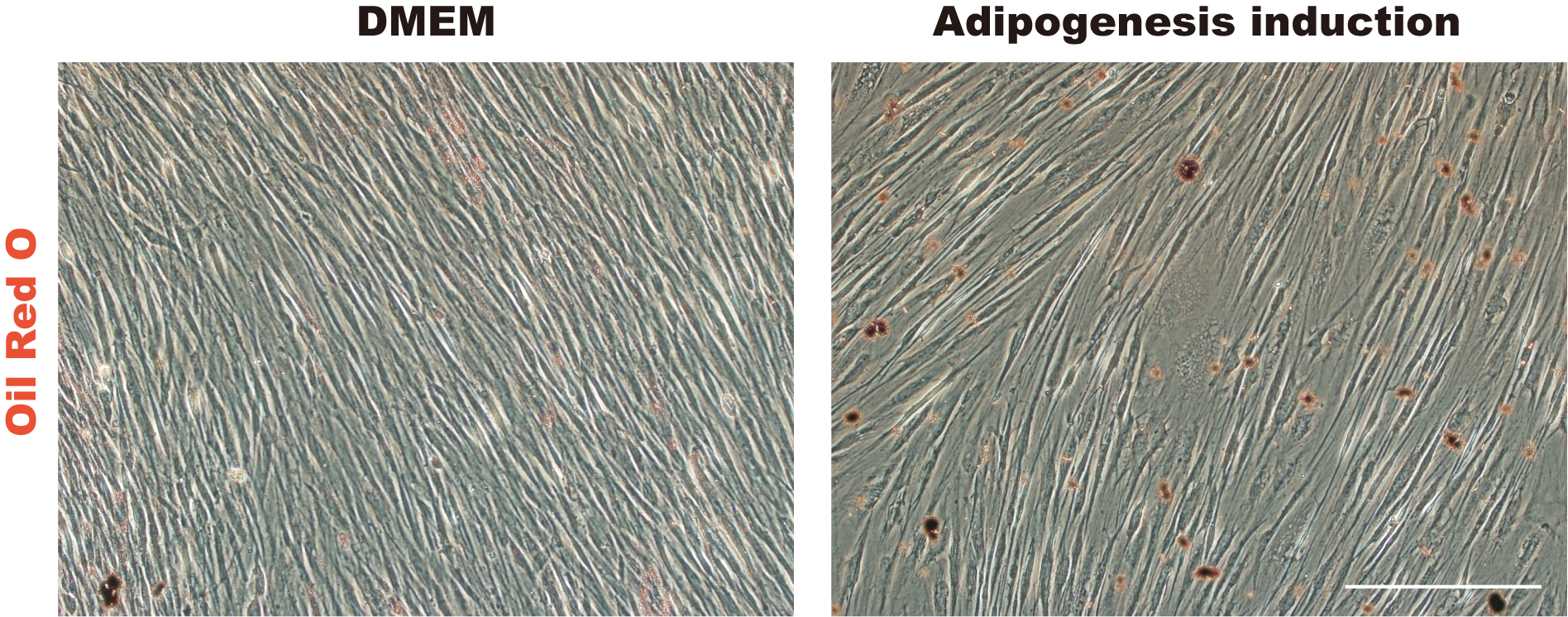

Supplement: Supplementary 1 — Figs. S1 to S11 [file research.1220.f1.zip › Figure S6.tif]

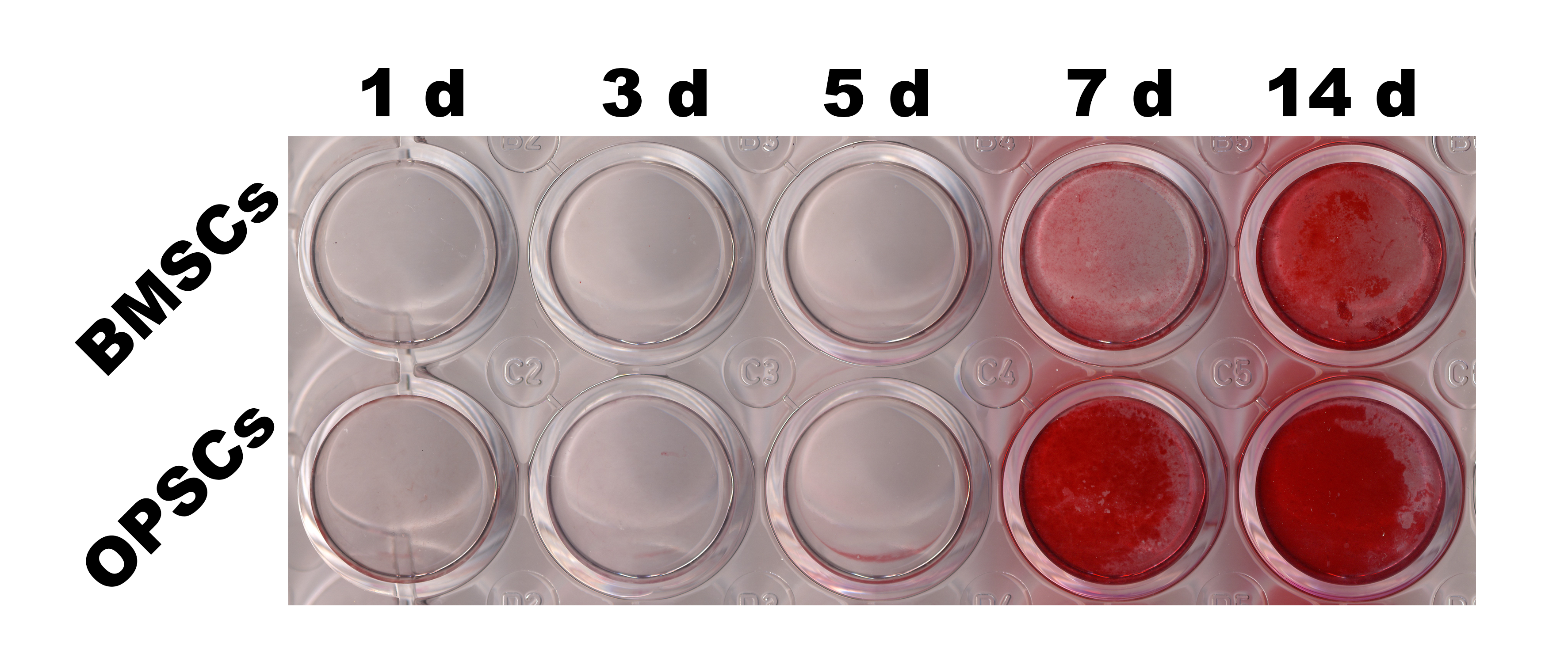

Supplement: Supplementary 1 — Figs. S1 to S11 [file research.1220.f1.zip › Figure s7.tif]

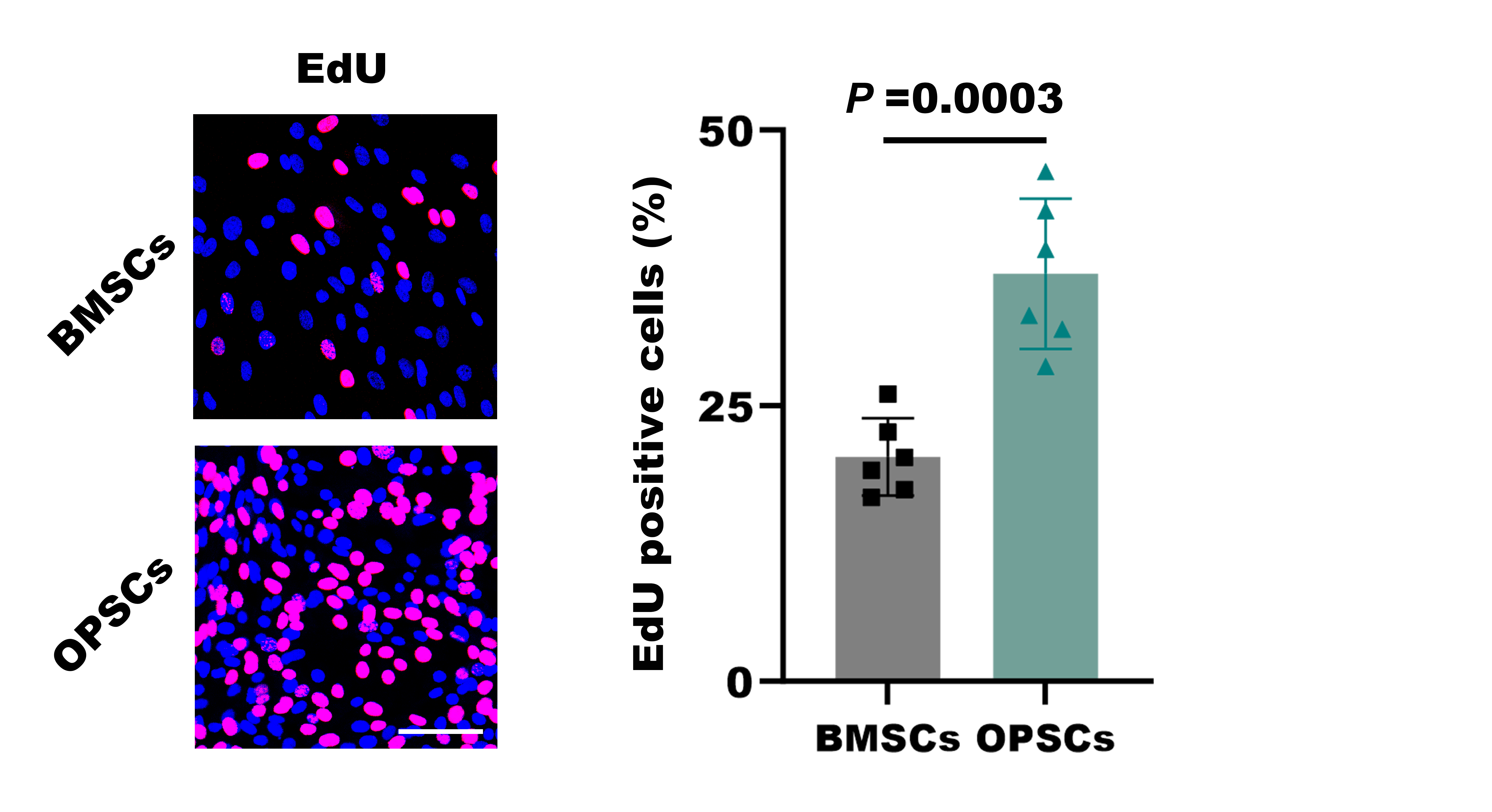

Supplement: Supplementary 1 — Figs. S1 to S11 [file research.1220.f1.zip › Figure s8.tif]

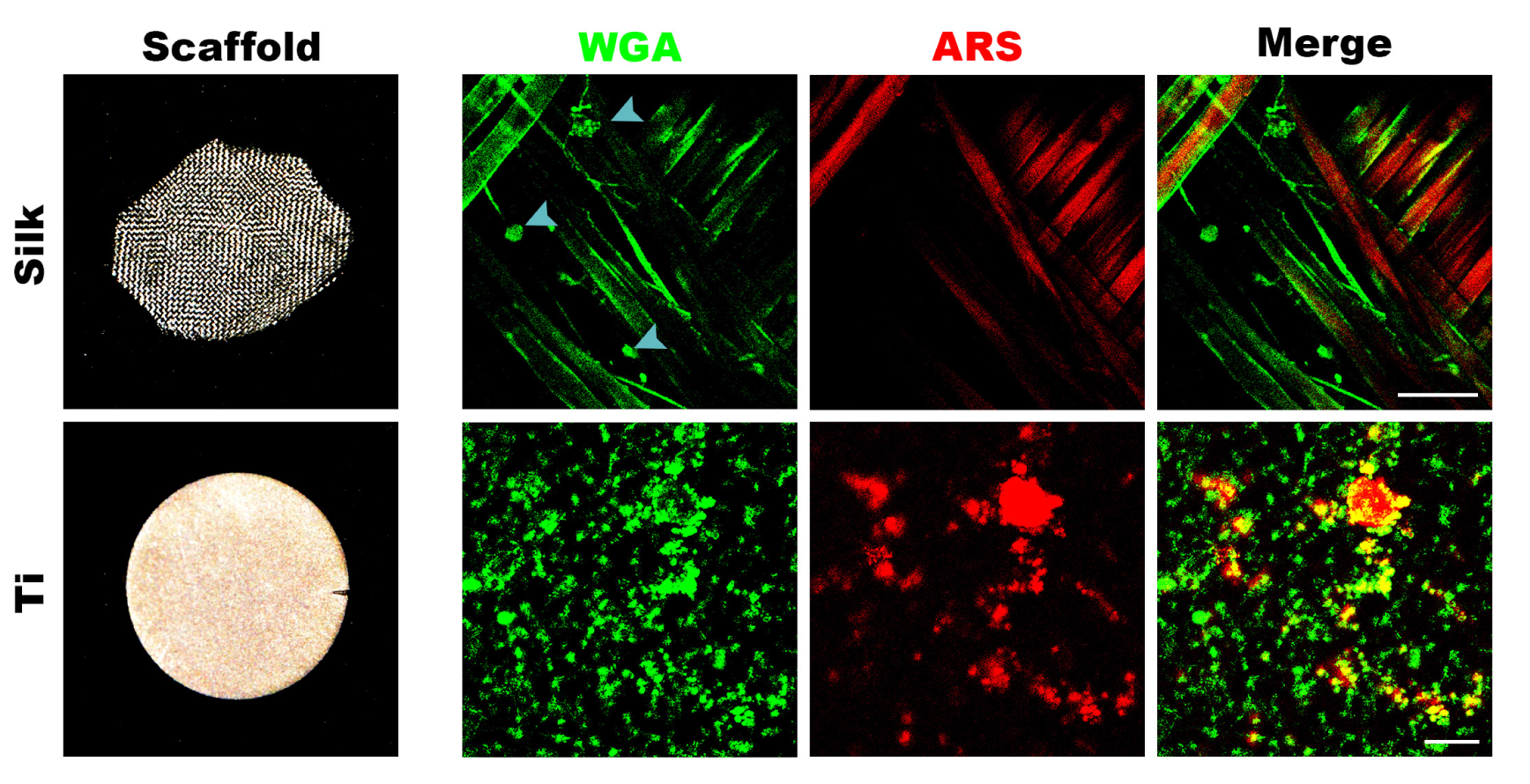

Supplement: Supplementary 1 — Figs. S1 to S11 [file research.1220.f1.zip › Figure S9.tif]
